# Dynamic role of the tether helix in PIP_2_-dependent gating of a G protein–gated potassium channel

**DOI:** 10.1085/jgp.201711801

**Published:** 2017-08-07

**Authors:** Emre Lacin, Prafulla Aryal, Ian W. Glaaser, Karthik Bodhinathan, Eric Tsai, Nidaa Marsh, Stephen J. Tucker, Mark S.P. Sansom, Paul A. Slesinger

**Affiliations:** 1 Fishberg Department of Neuroscience, Icahn School of Medicine at Mount Sinai, New York, NY; 2 Friedman Brain Institute, Icahn School of Medicine at Mount Sinai, New York, NY; 3 Department of Biochemistry, University of Oxford, Oxford, England, UK; 4 OXION Initiative in Ion Channels and Disease, University of Oxford, Oxford, England, UK; 5 Department of Physics, University of Oxford, Oxford, England, UK; 6 Novartis, Boston, MA

## Abstract

G protein–gated inwardly rectifying potassium (GIRK) channels control neuronal excitability in the brain and are implicated in several different neurological diseases. The anionic phospholipid phosphatidylinositol 4,5 bisphosphate (PIP_2_) is an essential cofactor for GIRK channel gating, but the precise mechanism by which PIP_2_ opens GIRK channels remains poorly understood. Previous structural studies have revealed several highly conserved, positively charged residues in the “tether helix” (C-linker) that interact with the negatively charged PIP_2_. However, these crystal structures of neuronal GIRK channels in complex with PIP_2_ provide only snapshots of PIP_2_’s interaction with the channel and thus lack details about the gating transitions triggered by PIP_2_ binding. Here, our functional studies reveal that one of these conserved basic residues in GIRK2, Lys200 (6′K), supports a complex and dynamic interaction with PIP_2_. When Lys200 is mutated to an uncharged amino acid, it activates the channel by enhancing the interaction with PIP_2_. Atomistic molecular dynamic simulations of neuronal GIRK2 with the same 6′ substitution reveal an open GIRK2 channel with PIP_2_ molecules adopting novel positions. This dynamic interaction with PIP_2_ may explain the intrinsic low open probability of GIRK channels and the mechanism underlying activation by G protein Gβγ subunits and ethanol.

## Abstract (precis)

G protein–gated inwardly rectifying potassium (GIRK) channels are activated by the phospholipid phosphatidylinositol 4,5 bisphosphate (PIP_2_). Using functional and computational experiments, Lacin et al. reveal that PIP_2_ interacts with the tether helix of the neuronal GIRK channel in a dynamic way.

## Introduction

G protein–gated inwardly rectifying potassium (GIRK or Kir3) channels are expressed in various regions of the brain, where they control the resting membrane potential and excitability of neurons (Lüscher and Slesinger, 2010). Mouse and human molecular genetic studies have indicated a role for GIRK channels in a variety of human disorders, including addiction, alcoholism, Down’s syndrome, and depression (for a review, see Mayfield et al., 2015). Similar to other inwardly rectifying potassium channels, activation of GIRK channels hyperpolarizes the membrane potential, reducing neuronal excitability. A large number of neurotransmitters (e.g., GABA, dopamine, glutamate, serotonin, acetylcholine, and opioid peptides) stimulate G protein–coupled receptors that directly activate GIRK channels (Ehrengruber et al., 1997; Lüscher et al., 1997; Scanziani, 2000; Wiser et al., 2006; Luján et al., 2009) via G protein Gβγ subunits (Logothetis et al., 1987; Reuveny et al., 1994; Wickman et al., 1994). In addition to G proteins, alcohol has been shown to directly activate GIRK channels (Aryal et al., 2009). However, GIRK channels remain mostly closed in the absence of activators. The structural mechanism underlying their low probability of opening in the absence of such activators remains poorly understood.

Four different GIRK subunits (GIRK1, GIRK2, GIRK3, and GIRK4) have been identified in mammals (Lesage et al., 1995; Inanobe et al., 1999; Wickman et al., 2000; Lüscher and Slesinger, 2010), and each is regulated by the membrane phospholipid phosphatidylinositol 4,5 bisphosphate (PIP_2_). Huang et al. (1998) showed that depletion of PIP_2_ prevents GIRK activation by Gβγ subunits, indicating that PIP_2_ is a cofactor for G protein regulation of GIRK channels. In fact, PIP_2_ is an essential cofactor for many different types of ion channels (Hille et al., 2015). Interestingly, the strength of the PIP_2_ interaction with the channel can determine the level of basal channel activity, which varies considerably among different inward rectifiers. For example, constitutively open inward rectifiers, such as Kir2 and Kir4 channels, bind PIP_2_ with relatively high affinity and have an open probability of close to 1 (Hibino et al., 2010). In contrast, Kir channels that are gated by G proteins, ATP, or ethanol (e.g., Kir3/GIRK, Kir1, and Kir6) exhibit lower relative affinities for PIP_2_ and a corresponding lower open probability, suggesting a correlation between the strength of the PIP_2_ interaction and open channel probability (Hibino et al., 2010). GIRK channel activators, such as ethanol and Gβγ subunits, appear to increase the relative affinity for PIP_2_, leading to an increase in the frequency and/or channel open time (Huang et al., 1998; Petit-Jacques et al., 1999; Bodhinathan and Slesinger, 2013). Conversely, ATP binding to Kir6/SUR channels leads to a reduction in PIP_2_ relative affinity (Baukrowitz et al., 1998).

Several laboratories have investigated what determines the relative strength of PIP_2_ association with Kir channels. Mutagenesis studies originally identified several conserved basic amino acids as well as some hydrophobic residues that influence the strength of PIP_2_ interaction with the channel (Zhang et al., 1999; Soom et al., 2001; Lopes et al., 2002). Accordingly, the interaction of PIP_2_ with GIRK channels could be converted from a weak to a strong interaction by introducing point mutations in the putative PIP_2_-binding site, resulting in large agonist-independent currents similar to Kir2 channels (Zhang et al., 1999; Zhou et al., 2001). These studies led to the proposal that positively charged basic amino acids form an essential part of the PIP_2_-binding site and that changes in the association of PIP_2_ with this region of the channel underlie gating of different inward rectifiers. However, how the nature of the interactions with these positively charged amino acids changes during gating is unknown.

Atomic resolution structures of GIRK2 and Kir2.2 channels in the presence or absence of PIP_2_ have provided structural details on the molecular interactions underlying PIP_2_-mediated gating of Kir channels (Whorton and MacKinnon, 2011, 2013). Inward rectifiers possess two gating structures, a G loop gate located at the apex of the cytoplasmic domain and a hydrophobic gate formed by amino acids in the pore-facing M2 transmembrane helices (i.e., helix bundle-crossing [HBC] gate; Lüscher and Slesinger, 2010). Both gates must move sufficiently to support K^+^ conduction through the channel pore. A cluster of four basic amino acids in a helical structure, referred to as the “tether helix” (also called the C-linker), appears to coordinate PIP_2_ (Whorton and MacKinnon, 2011, 2013). Curiously, these structural studies reveal only subtle differences in how PIP_2_ interacts with Kir2.2 and GIRK2 channels (Hansen et al., 2011; Whorton and MacKinnon, 2011, 2013), with the positive charge appearing to have a dominant role in PIP_2_ binding for both channels. Furthermore, the GIRK2 structure solved in the presence of PIP_2_ and Gβγ subunits is partially closed, as is the Kir2.2/PIP_2_ structure (Hansen et al., 2011; Whorton and MacKinnon, 2011, 2013), suggesting that the binding of PIP_2_ to an open channel may look entirely different from the current set of atomic resolution structures. Another limitation with ion channel structures is they represent a static channel and lack the dynamic interactions that occur during gating. Thus, it remains to be determined how the molecular interactions of PIP_2_ with the channel protein explain the large differences in resting basal channel activity and subsequent activation among different Kir channels. Here, using a combination of functional electrophysiological studies and molecular dynamics (MD) simulations, we sought to investigate how the highly conserved basic amino acids in the tether helix contribute to channel activation.

## Materials and methods

### Molecular biology

The following cDNAs, mouse GIRK2c (Kir3.2c), mouse Kir2.1, human m2R, enhanced yellow fluorescent protein (eYFP), and *Danio rerio* voltage-sensitive phosphatase (*Dr-Vsp*; gift from Y. Okamura, Osaka University, Osaka, Japan) were cloned in the mammalian expression vector pcDNA3.1 (ThermoFisher). Point mutations were introduced by site-directed mutagenesis (QuikChange II XL, Agilent Technology) and confirmed by automated DNA sequencing. GIRK2* was generated by replacing four native cysteines in GIRK2 (or Kir3.2) channels with thiol-unreactive amino acids (C65V, C190T, C221S, and C321V).

### Electrophysiology

Whole-cell patch-clamp recordings were performed 24 to 48 h after transfection (Bodhinathan and Slesinger, 2013). Borosilicate glass electrodes (Warner Instruments) with access resistances ranging from 3 to 6 MΩ were filled with an intracellular solution containing 130 mM KCl, 20 mM NaCl, 5 mM EGTA, 5.46 mM MgCl_2_ (1.5 mM free Mg^2+^), 2.56 mM K_2_ATP, 0.3 mM Li_2_GTP, and 10 mM HEPES (pH 7.4, ∼313 mOsm). The extracellular “20K” solution contained 20 mM KCl with 140 mM NaCl, 0.5 mM CaCl_2_, 2 mM MgCl_2_, and 10 mM HEPES (pH 7.4, ∼318 mOsm). 100 mM ethanol, 1-propanol, or 2-methyl-2,4-pentanediol (MPD) were added directly to the 20K solution. MTS-HE, MTS-Y, and MTS-F (Tocris) were dissolved in DMSO and diluted in 20K solution directly before application to cells. Whole-cell patch-clamp recordings were made as described previously (Bodhinathan and Slesinger, 2013).

### MD simulations

The crystal structure of the GIRK2 channel bound to a PIP_2_ head group was used for MD simulations (see supplemental Materials and methods). We used the Protein Data Bank (PDB): 4KFM structure for simulations, because this channel is in a preopen conformation (Whorton and MacKinnon, 2013). We compared the 3SYA and 4KFM structures and determined there is a small RMSD (Whorton and MacKinnon, 2011, 2013), with the largest movements in the LM loop and a 0.41 Å RMSD for the c-α in the PIP_2_ pocket, formed by residues 88–92 (from the slide helix), 192–203 (HBC gate and tether helix), and 62–64 (N-terminal b-loop). Four 1-stearoyl-2-arachidonoyl-*sn*-glycero-3-phosphoinositol 4,5 bisphosphate (18:0, 22:4 PIP_2_) molecules were aligned to the crystal structure of PIP_2_, one at each binding site. Gβγ subunits were not included in the calculations. The GIRK2-PIP_2_ system was embedded in a bilayer consisting of 202 1-palmitoyl-2-oleoyl-*sn*-glycero-3-phosphocholine (POPC; 16:0, 18:1 PC) lipids using the CHARMM-GUI membrane builder (Brooks et al., 2009; Wu et al., 2014). The protein–lipid system was then solvated with TIP3P water molecules and 150 mM KCl. The CHARMM36 force field was employed (Best et al., 2012). Additionally, five potassium ions were placed in the ion conduction pathway, and four sodium ions were placed in the sodium-binding site as seen in the crystal structures. A second system was set up with the same conditions but with a 6′Y mutation generated by in silico mutagenesis using CHARMM-GUI. The two systems (WT and 6′Y) were subjected to stepwise decreased restraint equilibration used in CHARMM-GUI membrane builder (Jo et al., 2007). The two systems (WT and 6′Y) were then equilibrated for 20 ns using positional restrain of 1,000 KJ/mol/nm^2^ on all Cα atoms of the protein before the 400-ns unrestrained production simulations. We then repeated the 400-ns production simulations using different initial velocities for both systems. Simulations were conducted using GROMACS 4.6 (Hess et al., 2008) with a 2 fs integration time step and visualized using VMD, Pymol (Humphrey et al., 1996), or Discovery Visualizer 4.0 (Accelrys).

### GIRK2 K^+^ flux assay

Mouse GIRK2 (containing amino acids 52–380) was expressed and purified in *Pichia pastoris* (a gift from R. Mackinnon, The Rockefeller University, New York, NY) as described previously (Whorton and MacKinnon, 2011; Glaaser and Slesinger, 2017). Purified GIRK2 channels were reconstituted into a lipid vesicle mixture containing POPE/POPG as described previously (Whorton and MacKinnon, 2011). For the K^+^ flux assay, vesicles were diluted 1:200 into flux buffer containing the H^+^-sensitive dye 9-amino-6-chloro-2-methoxyacridine (ACMA), as described previously (Whorton and MacKinnon, 2011; Glaaser and Slesinger, 2017). Fluorescence quenching was measured using a *Flexstation 3* microplate reader (Molecular Devices). Changes in fluorescence were monitored after the addition of the H^+^ ionophore *m*-chlorophenyl hydrazone (CCCP), followed by the addition of 30 µM diC_8_-lipids (PI, PI(4)P, PI(5)P, PI(3,4)P_2_, PI(4,5)P_2_, and PI(3,4,5)P_3_; Echelon). The K^+^ gradient was collapsed with 50 nM of the K^+^ ionophore valinomycin, allowing for determination of vesicle capacity at the end of the experiment. The “relative K^+^ flux” was calculated by measuring the decrease in fluorescence (i.e., quenching) 900 s after the addition of a vehicle or phospholipid. This decrease in fluorescence was normalized to the basal fluorescence before adding CCCP and to the maximal decrease in fluorescence following the addition of valinomycin (F_v_). The leakage flux for the proteoliposomes (no diC_8_) was subtracted to give the “fractional activation.”

### Statistical analyses

Pooled data are presented as mean ± SEM and evaluated for statistical significance (defined as P < 0.05 for all tests). An unpaired Student’s *t* test was used to compare data from two separate groups. One-way ANOVA was used for comparing three or more groups, followed by Bonferroni post hoc test for comparison between groups when applicable. Where indicated, n represents the number of cells from which the currents were recorded under each condition. All data were analyzed using Excel (Microsoft) and Prism (GraphPad).

### Online supplemental material

Additional details regarding the methods, along with supplemental data and videos, are provided online. Figs. S1–S6 contain additional analyses of the MD simulations, and Videos 1–4 are 400-ns videos of the WT and KY MD simulations, focusing on the PIP_2_ interaction in the PIP_2_ pocket and movement of the -2′F (F192) in the helix bundle crossing gate.

## Results

### Critical role for the 6′ lysine in the tether helix for PIP_2_-dependent gating

**Figure 1.**
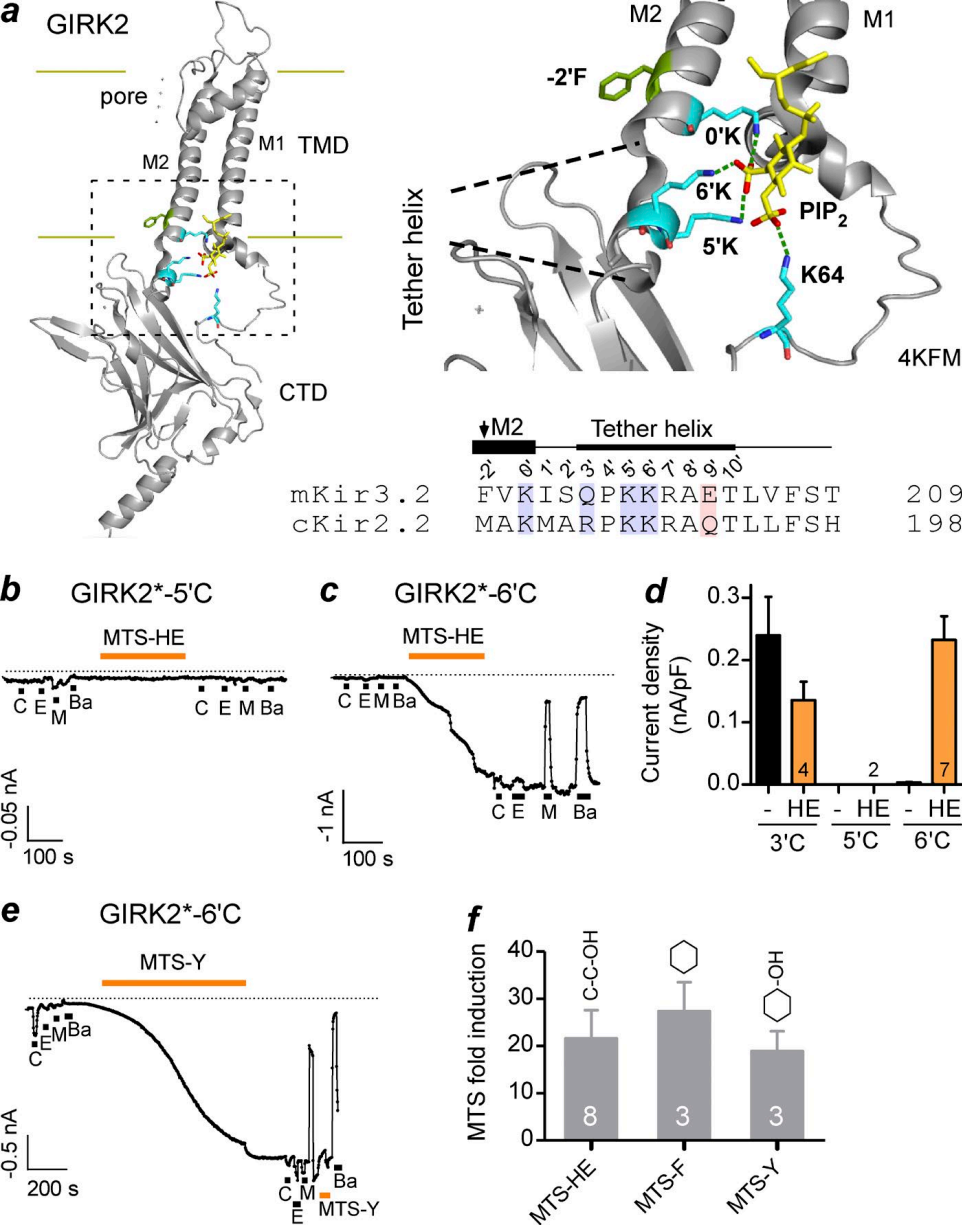
**The positive charge at 6’K is not essential for activation of GIRK2 channels**. (a) Model shows a single subunit of GIRK2 (PDB: 4KFM), highlighting the region involved in binding PIP_2_. Four lysines form hydrogen bonds with the 4′ and 5′ phosphates of PIP_2_. Amino acid sequences of the tether helix for mouse GIRK2 (Kir3.2) and chicken Kir2.2 are shown below with the proposed number system. Arrow indicates HBC. (b) Current trace for GIRK2*-5′C shows little effect of 5 µM carbachol (C), 100 mM ethanol (E), 100 mM MPD (M) or Ba^2+^ before or after exposure to 1 mM MTS-HE. Vh= −100 mV. (c) Current trace for GIRK2*-6′C shows large activation of basal current with 1 mM MTS-HE. Note the inhibition of MTS-HE–activated current with both Ba^2+^ and MPD. (d) Bar graph shows mean increase in basal current density (pA/pF) measured at −100 mV. (e) Current trace for GIRK2*-6′C shows large activation of basal current with 0.1 mM MTS-F. (f) Mean fold-induction of basal current with 0.1 mM MTS-HE, 0.1 mM MTS-F, and 0.1 mM MTS-Y for GIRK2*-6′C. Bars show mean ± SEM.

To better understand the structural mechanism underlying PIP_2_-dependent gating of GIRK channels, we evaluated the role of the positively charged, basic amino acids in the tether helix (Whorton and MacKinnon, 2011, 2013). To facilitate a comparison with the previous studies that have examined these highly conserved charged residues, we propose a numbering system whereby the first of these highly conserved residues (K194 in GIRK2) is defined as the 0′ position (0′K), K199 as 5′K, and K200 as 6′K (Figs. 1 a and S1 a).

In the GIRK2/PIP_2_/Gβγ atomic structure (PDB: 4KFM), which is considered to be in a preopen conformation (Whorton and MacKinnon, 2013), 0′K, 5′K, and 6′K in the tether helix form hydrogen bonds with the 5′ phosphate (5′-PO_4_) of PIP_2_ (Fig. 1 a). In the Kir2.2/PIP_2_ structure (PDB: 3SPI), the 0′K and 5′K form hydrogen bonds with the 5′-PO_4_, but the 6′K forms a hydrogen bond with the 4′-PO_4_ (Fig. S1 b; Hansen et al., 2011). Thus, the 6′K in the tether helix of GIRK2 and Kir2.2 interact with different phosphates on the inositol ring of PIP_2_. Given these variations in how PIP_2_ is coordinated in the GIRK2 and Kir2.2 structures, we hypothesized that this region of the tether helix may be important for regulating the PIP_2_-dependent gating of GIRK2 channels.

To test this idea, we used a strategy of chemical modification in which a single cysteine is introduced at 5′K or 6′K and is then probed with a membrane permeant MTS compound. This technique has the advantage of enabling the study of a channel before and after modification and has been used previously to probe gating structures of inwardly rectifying potassium channels (Guo et al., 2002; Lopes et al., 2002; Xiao et al., 2003; Bodhinathan and Slesinger, 2013). Here, we focused on the effect of MTS hydroxyethyl (HE), which would covalently attach a CH_2_-CH_2_-OH side chain to the sulfhydryl of the engineered cysteine, mimicking a serine/threonine type of amino acid substitution, i.e., a polar, uncharged residue. We introduced the cysteine into a GIRK2 channel that was modified to contain no other internal cysteines (referred to as GIRK2*). GIRK2* was shown previously to retain both Gβγ- and alcohol-dependent activation (Bodhinathan and Slesinger, 2013). Using whole-cell patch-clamp recordings from HEK293 cells transiently transfected with GIRK2*-5′C or GIRK2*-6′C cDNA, along with the m2 muscarinic receptor cDNA, we measured the basal, m2 muscarinic receptor–activated and alcohol-activated GIRK2 currents before and after MTS modification (Bodhinathan and Slesinger, 2013). In HEK293 cells expressing GIRK2*-5′C, there was little activation by carbachol or alcohol, and transfected cells did not show any appreciable Ba^2+^-sensitive basal currents. Bath application of MTS-HE (0.1–1 mM) for ∼5 min did not appear to alter the basal currents (Fig. 1 b; *n* = 2). Expression of GIRK2*-6′C in HEK293 cells also produced currents that were small and relatively insensitive to 5 µM carbachol or 100 mM alcohols (Fig. 1 c). In contrast to 5′C, however, bath application of 1 mM MTS-HE dramatically increased the amplitude of the Ba^2+^-sensitive basal current for GIRK2*-6′C channels (Fig. 1, c and d). The neighboring 3′C in the tether helix also showed no increase in basal current with MTS-HE (Fig. 1 d). For 6′C, the basal current density increased from −3.4 ± 0.8 pA/pF to −232.2 ± 38.0 pA/pF (*n* = 7) and was now inhibited by ∼90% with MPD (−89.5% ± 6.9%, *n* = 7) after modification with MTS-HE.

We next examined the effect of two other membrane-permeant MTS reagents, 0.01 mM MTS-phenylalanine (F) and 0.1 mM MTS-tyrosine (Y). Like MTS-HE, MTS-Y and MTS-F dramatically increased the Ba^2+^-sensitive basal currents by ∼20-fold (Fig. 1, e and f). These results demonstrate that the positive charge of 6′K is not essential for MTS activation of GIRK2*-6′C channels. In addition, MPD inhibited the MTS-activated basal currents from 60% to 100% after modification with MTS-F and MTS-Y (Figs. 1 e and S2). A large agonist-independent basal current and inhibition with MPD are features of a constitutively active GIRK2 and Kir2.1 channels (Aryal et al., 2009). These results demonstrate the Cys at the 6′ position is unable to support channel activation (i.e., little Ba^2+^-sensitive current), but modification with a larger uncharged side chain (e.g., Y or F) greatly activates the channel.

**Figure 2.**
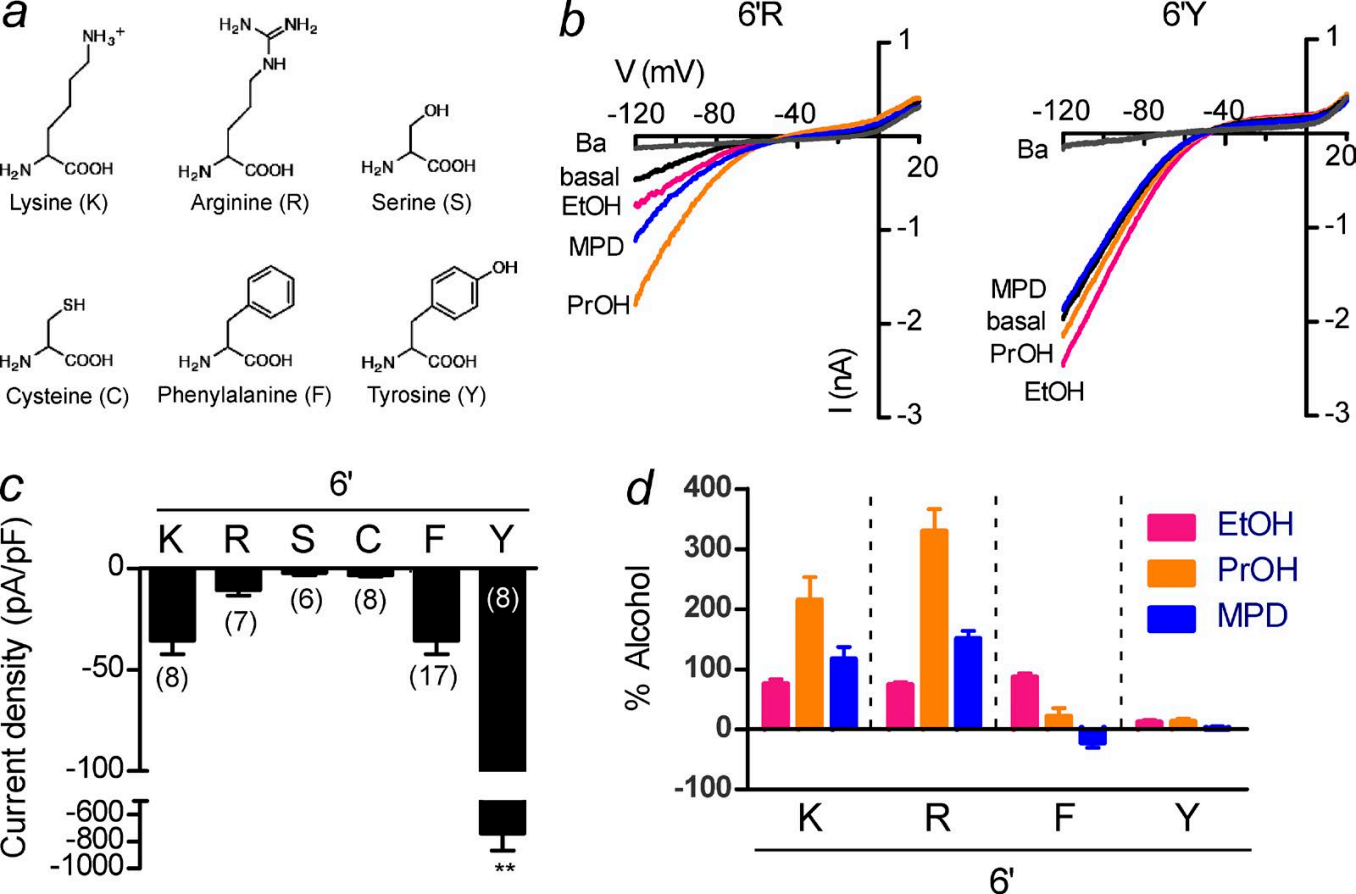
**A tyrosine substitution at 6′K increases agonist-independent basal current**. (a) Structures of amino acids introduced at 6′K in GIRK2. (b) Current–voltage plots show the change in inwardly rectifying current with different alcohols and Ba^2+^ for 6′R and 6′Y. (c) Bar graph shows change in current density with different substitutions at 6’K. Note the significantly larger current for 6′Y (**, P < 0.05). (d) Mean percentage change in current, relative to the Ba^2+^ basal, for 100 mM ethanol (EtOH), 100 mM 1-propanol (PrOH), or MPD (100 mM) with different amino acid substitutions at the 6′ position. Note the reduced alcohol responses for 6′Y (*n* = 8–18). Bars represent mean ± SEM.

With the MTS modification experiments, there are four cysteines per tetrameric channel that can be modified but the exact stoichiometry of modification is unknown. To study the effect of four substitutions per tetramer, we engineered a single point mutation at 6′K in GIRK2*. We predicted that a conserved charged substitution at this position (i.e., K200R [6′R]) would produce a channel with a small basal current and normal alcohol modulation, whereas a phenylalanine or tyrosine substitution would most closely mimic modification with MTS-F or MTS-Y (i.e., increase the basal current and change alcohol activation). Expression of GIRK2*-6′R produced channels with a small Ba^2+^-sensitive basal current, which was activated by ethanol, propanol, and MPD, similar to GIRK2* channels (Fig. 2, a–d). In contrast, mutation of this positively charged residue to a tyrosine (GIRK2*-6′Y) produced large Ba^2+^-sensitive basal currents that were relatively insensitive to alcohols, similar to MTS-Y modification of 6′C. Expression of GIRK2*-6′F produced Ba^2+^-sensitive basal currents that were inhibited by MPD, similar to MTS-F modification of 6′C, but the basal current was not significantly larger (Fig. 2, c and d). Collectively, these results suggest that a Tyr side chain at the 6′ position of the tether helix may strengthen the interaction with PIP_2_, leading to a large basal current and small alcohol response (Aryal et al., 2009).

**Figure 3.**
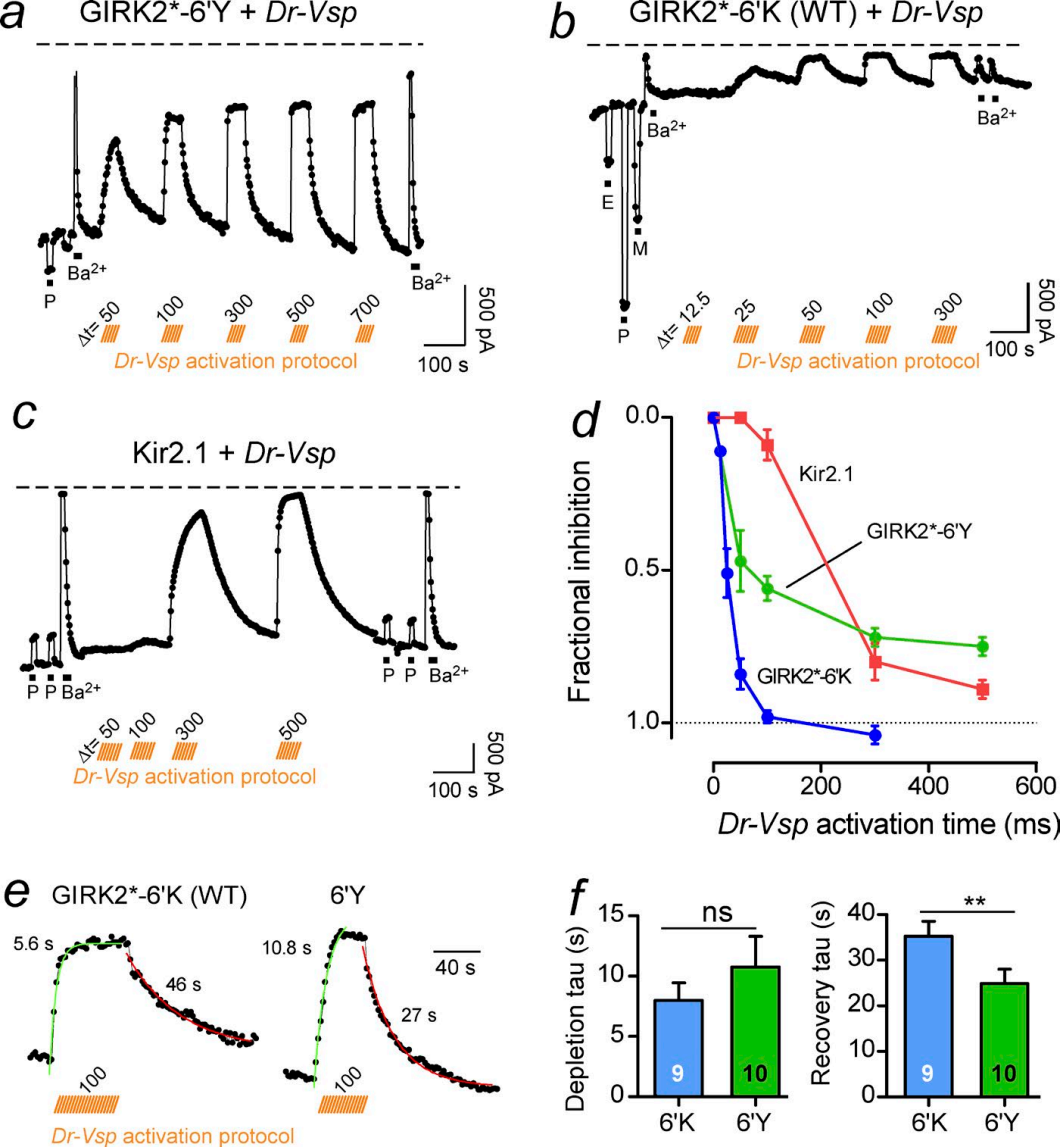
**GIRK2 6′Y channels display altered association for PIP_2_**. (a–c) The current is plotted as a function of time for a cell expressing *Dr-Vsp* with GIRK2*-6′Y (a), GIRK2*-6’K (WT; b), or Kir2.1 (c) channels. V_h_ = −120 mV. Voltage-dependent (+100 mV) activation times for *Dr-Vsp* are indicated by orange bars. 100 mM 1-propanol, 100 mM ethanol, or 100 mM MPD (P, E, and M, respectively) and 3 mM Ba^2+^ were applied before and after *Dr-Vsp* activation. (d) Fractional inhibition of steady-state basal current is plotted as a function of different *Dr-Vsp* activation times. Note the rank order of Kir2.1 > GIRK2*-6′Y > GIRK2* depletion time. *n* = 7–10. (e) Representative examples of GIRK current depletion and recovery after 100 ms activation of *Dr-Vsp* at +100 mV. V_h_ = −120 mV. Best-fit single exponentials with time constants are shown. (f) Mean tau (s) for depletion and recovery of current following Dr-Vsp–dependent depletion. **, P < 0.05; ns, not significant; Student’s *t* test. Bars represent mean ± SEM.

To probe for possible changes in the relative association of PIP_2_ with the channel, we studied the effect of reversibly depleting plasma membrane PIP_2_ on whole-cell Kir currents using the voltage-activated phosphatase *Dr-VSP* (Bodhinathan and Slesinger, 2013; Adney et al., 2015; Rjasanow et al., 2015). Previous studies showed that activation of *Dr-Vsp* by depolarizing the membrane to +100 mV for varying lengths of time produces a time-dependent, graded depletion of PIP_2_ and a corresponding reduction in Kir current (Bodhinathan and Slesinger, 2013). To examine the relative association of PIP_2_ with GIRK2*-6′Y channels, we coexpressed *Dr-Vsp* with 6′Y channels and studied the effect of varying length prepulses to +100 mV on the GIRK current measured at −120 mV (Fig. S3). Longer depolarization times led to more rapid and complete depletion of membrane-bound PIP_2_. Both 300 and 500 ms prepulses reduced the steady-state basal current, reaching a maximal inhibition of ∼70% of the Ba^2+^-sensitive basal current (0.7 fractional inhibition; Fig. 3, a and d). The 30% residual current could reflect tightly bound PIP_2_ or a change in PIP selectivity and was not examined further. In contrast, activation of *Dr-Vsp* with shorter prepulses (e.g., 50 or 100 ms) reduced ∼100% of the basal current for WT GIRK2*-6’K channels (Fig. 3, b and d). For comparison, we also measured the *Dr-Vsp* sensitivity of Kir2.1 channels, which are known to bind PIP_2_ with higher relative affinity (Huang et al., 1998; Sui et al., 1998). Expression of Kir2.1 produced large Ba^2+^-sensitive basal currents that were partially inhibited by 100 mM propanol (Fig. 3, c and d), similar to previous results (Kobayashi et al., 1999; Lewohl et al., 1999). Activation of *Dr-Vsp* for 50 or 100 ms produced little change in the basal current, whereas a 500 ms prepulse significantly reduced the Ba^2+^-sensitive basal currents by ∼90% (Fig. 3, c and d).

We next analyzed the rates of current depletion and recovery after *Dr-Vsp* activation by fitting the decay of current with a single exponential and determining the time constant (tau). These rates correlate with the differences in PIP_2_ affinity, where higher-affinity channels typically have a slow depletion and fast recovery (Bodhinathan and Slesinger, 2013; Adney et al., 2015; Rjasanow et al., 2015). The rate of recovery depends on both the relative affinity of the channel for PIP_2_ and the rate of resynthesizing membrane-bound PIP_2_. We assume the latter is similar when comparing different channels. We found that the tau for depletion was not significantly different for 6′K and 6′Y channels, but the tau for recovery was significantly faster for GIRK2*-6′Y (Fig. 3, e and f), 24.9 ± 3.2 s (*n* = 10) for 6′Y, and 35.8 ± 3.4 s (*n* = 9) for 6′K. This faster recovery for 6′Y supports the interpretation of a stronger PIP_2_ interaction (Bodhinathan and Slesinger, 2013; Adney et al., 2015; Rjasanow et al., 2015; Dai et al., 2016).

### MD simulations of PIP_2_-dependent gating

**Figure 4.**
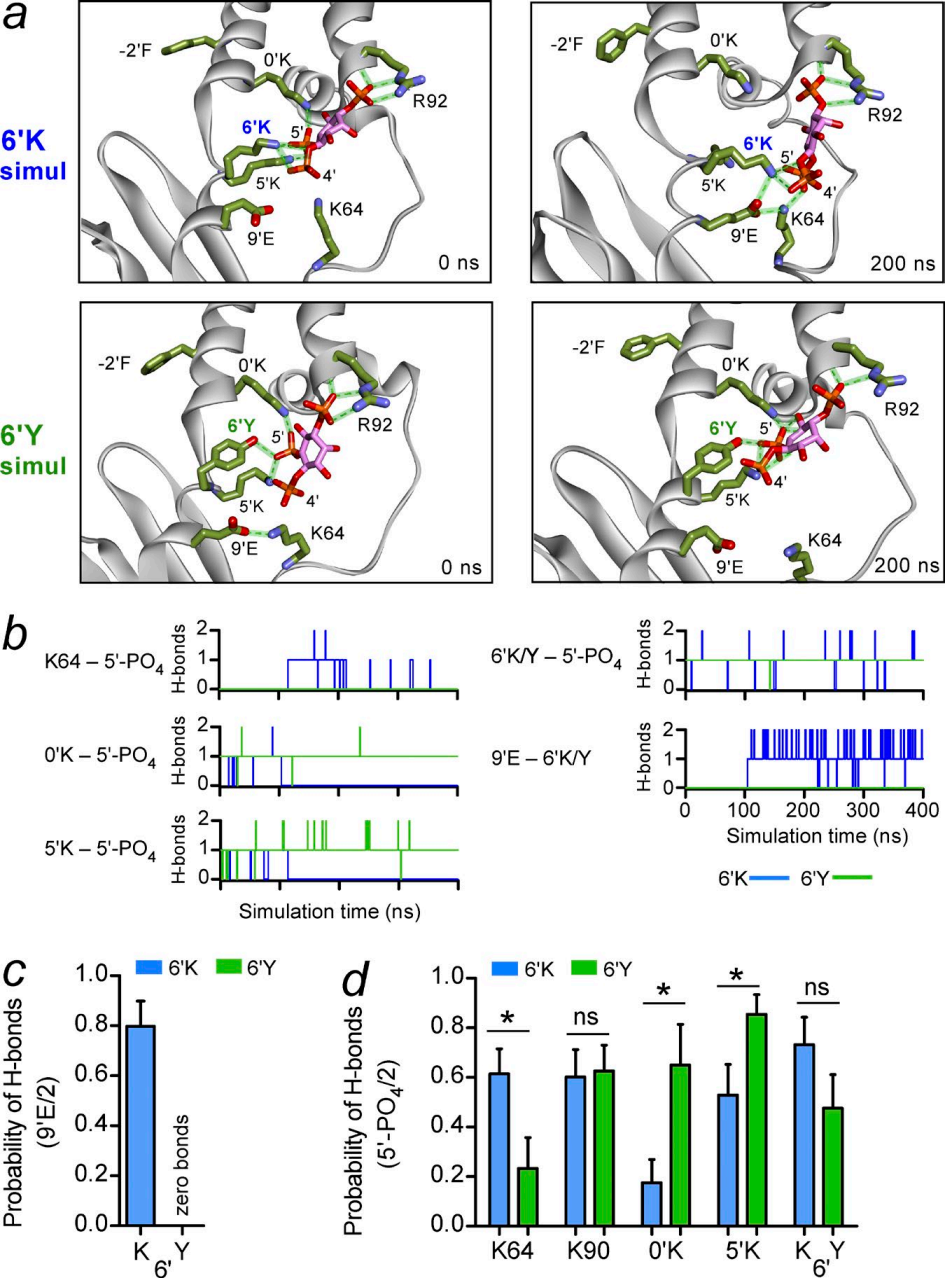
**Dynamic change in hydrogen bonding with PIP_2_ in GIRK2-6′K and GIRK2-6′Y MD simulations**. (a) Examples of GIRK2/PIP_2_ structures at the start and after 200 ns of MD simulations for GIRK2-6′K (WT) and GIRK2-6′Y channels. Note the 5′-PO_4_ of PIP_2_ moves away from starting position in the WT simulation. In contrast, the 5′-PO_4_ of PIP_2_ appears to engage PIP_2_ more deeply in the pocket in the 6′Y channel. One of four subunits is shown. (b) Each graph plots the number of hydrogen bonds during the 400-ns simulation time for the indicated pairs for WT GIRK2-6′K (blue) and GIRK2-′Y (green) for the PIP_2_ binding pockets shown in a. (c and d) Bar graphs show the mean relative probability of hydrogen bonds for all four subunits during the two 400-ns simulations for GIRK2-6′K (blue) and GIRK2-6′Y (green) channels. (c) Probability of hydrogen bonding between 9′E and 6′K/Y. (d) Probability of hydrogen bonding for K64, K90, 0′K, 5′K, and 6′K/Y with 5′-PO_4_ of PIP_2_ (of two maximal bonds). Note the number of hydrogen bonds increases with 0′K and 5′K but decreases for K64 with the 6′Y channel simulations. Bars represent mean ± SEM. *, P < 0.05; ns, not significant; Student’s *t* test.

To gain structural insights into how 6′Y might play a critical role in determining PIP_2_ interactions, we conducted atomistic MD simulations to investigate possible differences in the PIP_2_ pocket. GIRK2 (6′K; WT) or GIRK2 6′Y channels were embedded in a POPC bilayer containing four PIP_2_ lipids with their head groups aligned onto their crystallographic positions. Using a starting condition defined as a preopen conformation, as depicted in the 4KFM structure (Whorton and MacKinnon, 2013), we examined the dynamics of PIP_2_ interaction with the WT (6′K) or 6′Y channel in the absence of Gβγ subunits during 400-ns simulations (Fig. 4; see Materials and methods for details). During simulation of the WT channel, the side chain of 6′K appears flexible and adopts a downward orientation, away from the transmembrane domains (TMDs). Furthermore, the 5′ phosphate group of PIP_2_ also moves downward away from 0’K in the pocket, forming a new set of interactions (Fig. 4 a, top; and Videos 1 and 2). In the 6′Y channel simulations, on the other hand, the 6′Y side chain maintains an upward orientation (i.e., points toward the lipid bilayer), and the 5′-PO_4_ of PIP_2_ appears to be more stably bound in its canonical site (Fig. 4 a, bottom; and Videos 3 and 4). To characterize the dynamics of these interactions, we examined the frequency of hydrogen bond formation with the 5′-PO_4_ of PIP_2_ of one chain in the 6′K or 6′Y channel over the 400-ns simulation (Fig. 4 b). The time-dependent plots show that the 0′K and 5′K form hydrogen bonds with the 5′-PO_4_, and at ∼100 ns, the hydrogen bonds break as the PIP_2_ molecule appears to move away from its canonical binding site (Fig. 4 b, blue trace). Coincident with this change, the negatively charged glutamate at 203 (E203; 9′E) forms a hydrogen bond with 6′K, whereas K64 forms a hydrogen bond with 5′-PO_4_. In the 6′Y channel simulations, however, both 0′K and 5′K maintain stable hydrogen bonds with the 5′-PO_4_ throughout the simulation, in parallel with the 6′Y forming a stable hydrogen bond to the same phosphate (Fig. 4 b, green trace). Furthermore, the 9′E does not form any hydrogen bonds with 6′Y.

We repeated the 400-ns simulations (simulation 2) to determine the reproducibility, and similar results were observed in the second simulation (Fig. S5, simulation 2). To quantify these observations, we calculated the probability of hydrogen bond formation between the 1′, 4′, or 5′ phosphate of PIP_2_ and GIRK2 for all four pockets in both 400-ns simulations (Fig. 4, c and d; and Figs. S4 and S5). Interestingly, we found that the highest probability of hydrogen bonds with PIP_2_ occurs between the side chain and backbone amide of R92 with the 1′-PO_4_ in both WT 6′K and 6′Y simulations (Figs. S4 a and S5). This finding raises the possibility that R92, which is at the bottom of the M1 helix, serves as an anchor point for interactions of 1′-PO_4_ of PIP_2_ with the channel, whereas the 4′-PO_4_ and 5′-PO_4_ interact more dynamically with the tether helix and N-terminal hairpin.

The probability of hydrogen bonds with the 5′-PO_4_ of PIP_2_ significantly increased for the 0′K and 5′K in the 6′Y channel simulation (Fig. 4 d). Concurrent with these changes, the probability of hydrogen bonds between K64 and 5′-PO_4_ decreased, whereas K64 hydrogen bonds with the 5′K increased in the 6′Y channel simulation (Figs. 4 d and S4 b). The probability of hydrogen bonds between the 4′-PO_4_ of PIP_2_ and K64 was unchanged but increased with the 5′K in the 6′Y channel simulation (Fig. S4 b). Furthermore, the 6′Y does not hydrogen bond with 9′E in the 6′Y simulation, allowing only hydrogen bond interactions with 5′-PO_4_ (Fig. 4, c and d). Collectively, the 6′Y simulations appear to show a strengthening of the interaction of the 5′-PO_4_ of PIP_2_ with the 0′K and 5′K, limiting the downward movement of PIP_2_ away from the channel’s PIP_2_-binding pocket (Fig. 4, a–d).

The MD simulations also revealed an important interaction of the 6′K with the 9′E, as the 6′K adopts a downward orientation (Fig. 4 a). The positively charged 6’K forms a significant number of hydrogen bonds with 9’E that are absent in the 6’Y channel (Fig. 4 c). The 9′E also forms hydrogen bonds with K64 in the MD simulations (Fig. S4 c). In the 6′Y mutant channels, however, the number of hydrogen bonds significantly decreases between K64 and the 5′-PO_4_ of PIP_2_ (Fig. 4 d). Thus, a trifecta of hydrogen bond interactions among 6′K, 9′E, and K64 may determine the extent of hydrogen bonding between PIP_2_ and the 0′K and 5′K sites in the channel. Collectively, the MD simulations provide a tenable model for how 6′Y might promote the channel to interact more strongly with PIP_2_; anchored by the 1′-PO_4_ at R92, the tyrosine substitution (6′Y) supports movement of PIP_2_ deeper into the pocket toward the 0′K and 5′K.

### In vitro assessment of 5′-PO_4_ in activating GIRK2 channels

**Figure 5.**
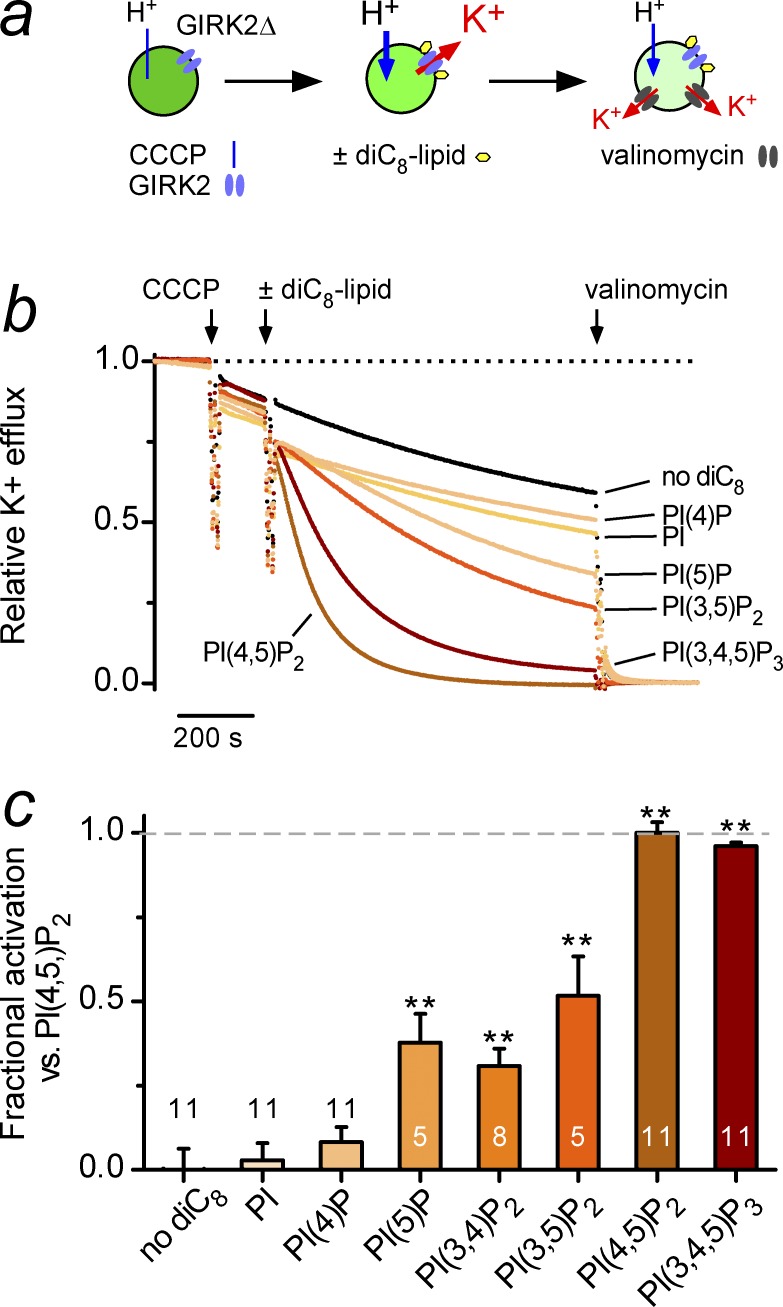
**PI(5)P is sufficient to activate GIRK2 channels in proteoliposomes**. (a) Cartoon shows design of fluorescence-based K^+^ flux assay with GIRK2-containing liposomes. (b) Normalized mean traces of K^+^ flux for GIRK2 with acute application of CCCP, 30 µM of the indicated diC_8_ phosphoinositides (blue), and valinomycin. SEM bars are omitted for clarity. (c) Bar graph shows steady-state response of each phosphoinositide normalized to the response with PI(4,5)P_2_ and after subtracting basal flux (e.g., “Fractional activation vs. PI(4,5)P_2_”). Bars represent mean ± SEM. **, P < 0.05 ANOVA with Bonferroni post hoc test versus control (no diC_8_).

The MD simulations indicate a prominent role for 5′-PO_4_ of PIP_2_ in GIRK2 channel gating. To test the role of the 5′-PO_4_ in channel activation, we examined effect of different soluble forms of phosphoinositides (e.g., diC_8_-PI(4,5)P_2_) on GIRK2 activation using purified GIRK2 channels reconstituted into proteoliposomes (Whorton and MacKinnon, 2011; Glaaser and Slesinger, 2017). Potassium flux through GIRK channels was monitored via a fluorescence-based K^+^ flux assay (Fig. 5 a; Whorton and MacKinnon, 2011). With GIRK2-containing liposomes loaded with the pH-sensitive ACMA dye, the addition of the proton ionophore CCCP results in quenching of the ACMA dye if GIRK2 channels are open (i.e., H^+^ enters via CCCP if K^+^ can exit the proteoliposome; Fig. 5 a; Whorton and MacKinnon, 2013). As expected, diC_8_-PI(4,5)P_2_, the soluble form of the predominant phosphoinositide found in the plasma membrane (Hille et al., 2015), produced maximal activation of purified GIRK2 channels (Fig. 5, b and c). PI(3,4,5)P_3_ elicited a K^+^ flux comparable to that of PI(4,5)P_2_, whereas PI(3,5)P_2_ and PI(3,4)P_2_ produced an intermediate K^+^ flux (Fig. 5 b). As shown previously (Ho and Murrell-Lagnado, 1999; Rohács et al., 1999), PI(4)P, and PI did not increase GIRK2 activity (Fig. 5, b and c). However, we found that application of PI(5)P alone activates GIRK2 channels (Fig. 5 c). To quantify these changes, we measured the steady-state flux and normalized to the maximal response with PI(4,5)P_2_. Indeed, PI(5)P significantly enhanced GIRK2 activity (Fig. 5 c). These data demonstrate that the two phosphates in the 4′ and 5′ position of PIP_2_ are necessary for complete channel activation but that the 5′-PO_4_ of PIP_2_ is sufficient to partially activate GIRK2 channels.

### MD simulations reveal conformational changes in the HBC gate

**Figure 6.**
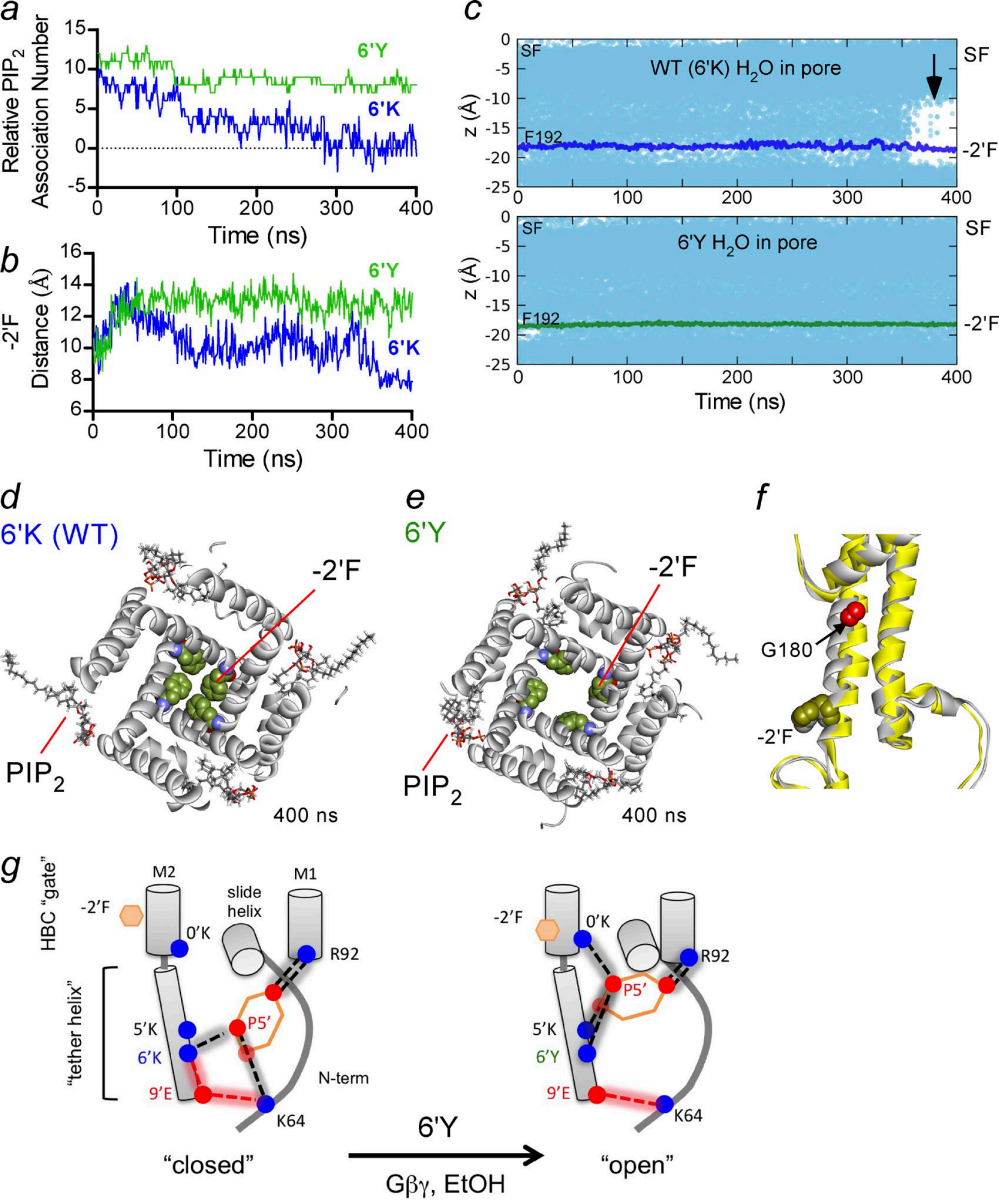
**MD simulations of GIRK2 6′Y reveal dynamic opening of the channel at the HBC gate**. (a) The relative PIP_2_ association number (see Materials and methods) is plotted as a function of simulation time for WT (6′K, blue) and 6′Y (green) channels. (b) The cross-distance diameter of the pore at the HBC, measured between the center of mass of F192 (−2′F) side chain, is plotted as a function of simulation time. (c) The water molecule density in the conduction pathway, moving in the z plane from the selectivity filter (0 Å) to the bottom of the M2 transmembrane domain (approximately −25 Å), is plotted as a function of the simulation time for WT (top) and 6′Y (bottom) channels. Note the loss of water around the region of the −2′F in WT channels (arrow). (d and e) Structural view of the HBC at 400 ns for GIRK2-WT (6′K; d) and GIRK2-6′Y (e). Note how PIP_2_ is bound loosely for WT and more tightly for 6′Y. (f) Superimposition of one subunit in 6′K (gray) and 6′Y (yellow) showing movement of M2 relative to G180 in 6′Y (red). (g) Cartoon summarizes hydrogen bond interactions for PIP_2_ and GIRK2 for new PIP_2_-closed and PIP_2_-open conformations based on MD simulations. EtOH, ethanol.

To determine whether PIP_2_ channel interactions observed in WT (6′K) and 6′Y channel simulations correlate with gating, we examined the time-dependent changes of PIP_2_ in all four PIP_2_ pockets at the level of F192 (-2’F), which forms the HBC gate. To quantify the relative association of PIP_2_ with all four pockets of the tetrameric channel during the simulation, we calculated the total number of the hydrogen bonds for 0′K–5′-PO_4_, 5′K–5′-PO_4_, and 6′K–5′-PO_4_ and subtracted the number of hydrogen bonds for non-PIP_2_ interactions (i.e., 6′K–9′E). This relative PIP_2_ association number provides an estimate of the extent of PIP_2_ interaction, where a high number indicates a stronger 5′-PO_4_ interaction within its binding site. Over the 400 ns, the relative PIP_2_ association number is ∼10 for 6′Y (Fig. 6 a). In contrast, this number decreases toward zero for the WT channel.

To correlate these effects with channel opening, we calculated the mean diameter across the pore at the HBC, F192 (−2′F). For the beginning of the WT 6′K simulation, the diameter at the HBC is ∼10 Å and decreases to <8 Å by the end of the simulation (Fig. 6 b). Coincident with the reduction in the PIP_2_ association number with WT channels, water density decreases in the pore around the HBC gate (i.e., “dewetting” occurs; Aryal et al., 2014), consistent with a closed channel (Fig. 6 c, arrow). In contrast, the diameter of 6’Y channel increases from 10 Å to ∼13 Å (Fig. 6 b), indicative of an open channel or a channel moving toward an open state. Furthermore, the M2 helix appears to move around G180, a site previously implicated in channel gating (Meng et al., 2012), in the 6′Y simulations (Fig. 6 f). These differences in the diameter of the pore are readily observed in the cross-sectional view of the channel at the HBC at the end of the 400-ns stimulation (Fig. 6, d and e). Therefore, as the channel transitions from a low to high PIP_2_ association number, the gate expands to ∼13 Å, and hydration of the pore occurs (Aryal et al., 2014), suggesting an open channel. The increase in water density in the pore around the HBC gate in the 6′Y channel, compared with WT, was also observed in the second 6′Y simulation (Fig. S6). Collectively, the MD simulations of WT-6′K and 6′Y channels reveal dynamic interactions of PIP_2_ within its binding pocket that are important for unique PIP_2_-dependent gating characteristics of GIRK2 channels.

## Discussion

Although it has been known for nearly 20 yr that PIP_2_ is required for activation of GIRK channels (Huang et al., 1998; Sui et al., 1998), the structural details of how the association of PIP_2_ with GIRK channels leads to channel activation remain poorly described. The initial crystal structures of Kir2.2 and GIRK2 channels provided snapshots of how PIP_2_ binds to Kir channels, implicating positively charged, basic amino acids in the tether helix, the M2 TMD, and the N-terminal domain in the binding of one PIP_2_ molecule (Hansen et al., 2011; Whorton and MacKinnon, 2011, 2013). Indeed, a comparison of the amino acids in the tether helix among different Kir channels reveals a high degree of conservation among these basic residues (Fig. S1 a). However, atomic resolution structures are static and lack the dynamic interactions of ligands associating with the channel and inducing gating conformations. In the current study, we combined functional studies with MD simulations to provide evidence for a dynamic balance of hydrogen bonds between the channel and PIP_2_, with the 6′K in the tether helix serving a unique role in regulating gating by PIP_2_.

Previous functional studies and mutagenesis experiments revealed some of the first clues on the mechanism of PIP_2_-dependent gating of Kir channels. Comparing the rates of current rundown while depleting PIP_2_ revealed two important features of PIP_2_-Kir gating. First, Kir channels differ in their apparent affinity for PIP_2_, whereby GIRK channels exhibit an apparent PIP_2_ affinity that is significantly lower than that for Kir2 channels (Huang et al., 1998; Sui et al., 1998). Second, the apparent affinity of GIRK channels for PIP_2_ increases in the presence of Gβγ subunits (Huang et al., 1998) or alcohol (Bodhinathan and Slesinger, 2013). Hypothesizing that negatively charged phosphates on PIP_2_ associate with positively charged basic residues in the channel, Lopes et al. systematically mutated basic residues in Kir2.1 and studied the effect of PIP_2_ antagonists (e.g., anti-PIP_2_ antibody or polylysine) on current levels. In the tether helix of Kir2.1, mutation of K188 to Cys (6′C) significantly reduced the association with PIP_2_ (Lopes et al., 2002). Modification of the 6′C with MTSEA, however, restored the inward current and PIP_2_ sensitivity, suggesting modification with the partial charge was sufficient to support gating. The 6′Q substitution, on the other hand, did not produce a functionally conducting channel (Lopes et al., 2002). Similarly, Soom et al. (2001) demonstrated that a 6′Q in Kir2.1 reduced the currents and PIP_2_ binding. Together, these results suggest that the positive charge at the 6′K position is important for coordinating PIP_2_ in Kir2.1.

In contrast, we find that positive charge at the 6′ amino acid in the tether helix is not essential for PIP_2_-dependent gating. Chemical modification with MTS-HE, MTS-F, and MTS-Y all increased the basal current. Furthermore, the conservative Arg substitution at the 6′K produced little change in the basal current in contrast to the Tyr substitution, which significantly enhanced the basal current. In addition, the 6′Y in GIRK2 appeared to increase the relative affinity for PIP_2_, primarily affecting the recovery rate after PIP_2_ depletion. Consistent with this finding, a glutamine substitution in Kir1.1 at K187 (6′Q) also appeared to strengthen the PIP_2_ interaction, whereas the neighboring 7′Q substitution (R188Q) dramatically reduced PIP_2_ association (Lee et al., 2016). Collectively, these findings raise the possibility that 6′K serves a unique role in GIRK2 as compared with that in Kir2.1 channels. Other differences exist between Kir2 and GIRK channels. Kir2 channels possess a secondary anionic phospholipid-binding site, which is absent in GIRK channels (Lee et al., 2013). Lastly, the cytoplasmic domain in the apo structure of Kir2.2 (i.e., no PIP_2_) is not engaged with the TMDs, whereas it remains fully engaged in the apo GIRK2 structure (Tao et al., 2009; Hansen et al., 2011; Whorton and MacKinnon, 2011).

In the atomic resolution structures of GIRK2-PIP_2_ and GIRK2-PIP_2_/Gβγ, which are proposed to be in a closed and preopen conformation, respectively, the 6′K forms a hydrogen bond with the 5′-PO_4_ of PIP_2_ in both structures (Whorton and MacKinnon, 2011, 2013). Similarly, MD simulations with KirBac3.1/GIRK1 channels showed that 5′K and the 6′K strongly interact with PIP_2_ in both the constricted and dilated forms (Meng et al., 2012). Together, these studies suggest the electrostatic association of 6′K with the negatively charged phosphate may have a more complex role than simply stabilizing PIP_2_ in the pocket. Our MD simulations confirmed that the 6′K (WT) forms hydrogen bonds with the 5′-PO_4_, but they also revealed that this interaction is dynamic. When the 6′K side chain points toward the TMDs (Fig. 6 g), the 5′-PO_4_ of PIP_2_ hydrogen bonds more tightly with 0′K and 5′K. Based on the functional studies of 6′Y mutation in GIRK2, which showed increased basal current and stronger interaction with PIP_2_, we also performed MD simulations with the 6′Y channel. Here, 6′Y stabilizes this upward orientation, increasing the probability of hydrogen bond formation for the 0′K and 5′K with the 5′-PO_4_ while decreasing the probability of hydrogen bond formation between K64 and the 5′-PO_4_ of PIP_2_. The movement of 6′K toward the cytoplasm and its subsequent interaction with E203 (9′E) may destabilize the interaction of 0′K and 5′K with the 5′-PO_4_ of PIP_2_, leading to closure of the HBC gate (Fig. 6 g) and perhaps explaining the low open channel probability for GIRK channels. K64 in the N-terminal domain may also contribute to PIP_2_-dependent gating of GIRK channels by hydrogen bonding with 9′E. It is possible GIRK channel activators, like Gβγ and ethanol, promote the interaction of K64 with 9′E and allow the 5′-PO_4_ to engage more strongly with the 0′K (Fig. 6 g). Interestingly, the homologous amino acid in Kir2.2 is a glutamine, supporting a possible unique role for K64 in GIRK channels.

The MD simulations and functional studies also highlight an important role for the 5′-PO_4_ of PIP_2_ to open GIRK2 channels. Consistent with this, we found that PI(5)P alone is sufficient to partially activate GIRK2 channels in reconstituted bilayers, which contrasts with its lack of effect on Kir2.1 channels (D’Avanzo et al., 2010). Furthermore, activation of *Dr-Vsp*, which dephosphorylates the 5′-PO_4_ of PIP_2_ to produce PI(4)P (Rjasanow et al., 2015), completely inhibits GIRK channel activity. Although PI(5)P is capable of activating, it is not present at high levels in the plasma membrane.

Importantly, the MD simulations suggest new PIP_2_-bound states that appear to capture a transition state on the path toward opening, an event that appears difficult to observe in any of the Kir channel crystal structures (Hansen et al., 2011; Whorton and MacKinnon, 2011, 2013). Recently, Meng et al. (2016) successfully modeled an open state of a chimeric GIRK1 channel using an M2-helix mutation, M170P. In these MD simulations, the M1 and M2 helices rotate counterclockwise, with a significant bending at G169, leading to a widening of the pore at the HBC gate (−2′F) that is sufficient to support K^+^ permeation. Interestingly, the binding mode of PIP_2_ did not change between the M170P-dilated and WT simulations. With the 6′Y MD simulations, however, we observe a similar movement of the M2 helix near G180, a widening of the HBC, and a tighter interaction of the 5′-PO_4_ of PIP_2_ with 0′K and 5′K of the channel. Additional mutagenesis experiments will be needed to fully validate this MD model of an open channel.

In conclusion, our experiments demonstrate that the highly conserved positive charge of the 6′K in GIRK2 supports a dynamic interaction with PIP_2_ that reduces the likelihood of channel opening in the absence of other activators (e.g., Gβγ or ethanol). This model may explain the intrinsically low open probability of GIRK channels and perhaps how the direct binding of physiologically relevant activators, such as Gβγ or ethanol, can modify these interactions to produce robust channel activation. It will be interesting to determine whether any particular features of this mechanism are involved in the regulation of channel activity in other types of Kir channel or whether this remains unique to the GIRK family.

## Supplementary Material

Supplemental Materials (PDF)

Video 1

Video 2

Video 3

Video 4

## References

[bib1] Adney S.K., Ha J., Meng X.Y., Kawano T., and Logothetis D.E. 2015 A critical gating switch at a modulatory site in neuronal Kir3 channels. J. Neurosci. 35:14397–14405. 10.1523/JNEUROSCI.1415-15.2015 26490875 PMC4683693

[bib2] Aryal P., Dvir H., Choe S., and Slesinger P.A. 2009 A discrete alcohol pocket involved in GIRK channel activation. Nat. Neurosci. 12:988–995. 10.1038/nn.2358 19561601 PMC2717173

[bib3] Aryal P., Abd-Wahab F., Bucci G., Sansom M.S., and Tucker S.J. 2014 A hydrophobic barrier deep within the inner pore of the TWIK-1 K2P potassium channel. Nat. Commun. 5:4377 10.1038/ncomms5377 25001086 PMC4102122

[bib4] Baukrowitz T., Schulte U., Oliver D., Herlitze S., Krauter T., Tucker S.J., Ruppersberg J.P., and Fakler B. 1998 PIP_2_ and PIP as determinants for ATP inhibition of K_ATP_ channels. Science. 282:1141–1144. 10.1126/science.282.5391.1141 9804555

[bib5] Best R.B., Zhu X., Shim J., Lopes P.E., Mittal J., Feig M., and Mackerell A.D. Jr 2012 Optimization of the additive CHARMM all-atom protein force field targeting improved sampling of the backbone φ, ψ and side-chain χ(1) and χ(2) dihedral angles. J. Chem. Theory Comput. 8:3257–3273. 10.1021/ct300400x 23341755 PMC3549273

[bib6] Bodhinathan K., and Slesinger P.A. 2013 Molecular mechanism underlying ethanol activation of G-protein-gated inwardly rectifying potassium channels. Proc. Natl. Acad. Sci. USA. 110:18309–18314. 10.1073/pnas.1311406110 24145411 PMC3831446

[bib7] Brooks B.R., Brooks C.L. III, Mackerell A.D. Jr., Nilsson L., Petrella R.J., Roux B., Won Y., Archontis G., Bartels C., Boresch S., 2009 CHARMM: The biomolecular simulation program. J. Comput. Chem. 30:1545–1614. 10.1002/jcc.21287 19444816 PMC2810661

[bib8] D’Avanzo N., Cheng W.W., Doyle D.A., and Nichols C.G. 2010 Direct and specific activation of human inward rectifier K+ channels by membrane phosphatidylinositol 4,5-bisphosphate. J. Biol. Chem. 285:37129–37132. 10.1074/jbc.C110.186692 20921230 PMC2988318

[bib9] Dai G., Yu H., Kruse M., Traynor-Kaplan A., and Hille B. 2016 Osmoregulatory inositol transporter SMIT1 modulates electrical activity by adjusting PI(4,5)P2 levels. Proc. Natl. Acad. Sci. USA. 113:E3290–E3299. 10.1073/pnas.1606348113 27217553 PMC4988571

[bib10] Ehrengruber M.U., Doupnik C.A., Xu Y., Garvey J., Jasek M.C., Lester H.A., and Davidson N. 1997 Activation of heteromeric G protein-gated inward rectifier K^+^ channels overexpressed by adenovirus gene transfer inhibits the excitability of hippocampal neurons. Proc. Natl. Acad. Sci. USA. 94:7070–7075. 10.1073/pnas.94.13.7070 9192693 PMC21286

[bib11] Glaaser I.W., and Slesinger P.A. 2017 Dual activation of neuronal G protein-gated inwardly rectifying potassium (GIRK) channels by cholesterol and alcohol. Sci. Rep. 7:4592 10.1038/s41598-017-04681-x 28676630 PMC5496853

[bib12] Guo Y., Waldron G.J., and Murrell-Lagnado R.D. 2002 A role for the middle C terminus of G-protein-activated inward rectifier potassium channels in regulating gating. J. Biol. Chem. 277:48289–48294. 10.1074/jbc.M207987200 12376541

[bib13] Hansen S.B., Tao X., and MacKinnon R. 2011 Structural basis of PIP2 activation of the classical inward rectifier K+ channel Kir2.2. Nature. 477:495–498. 10.1038/nature10370 21874019 PMC3324908

[bib14] Hess B., Kutzner C., van der Spoel D., and Lindahl E. 2008 GROMACS 4: Algorithms for highly efficient, load-balanced, and scalable molecular simulation. J. Chem. Theory Comput. 4:435–447. 10.1021/ct700301q 26620784

[bib15] Hibino H., Inanobe A., Furutani K., Murakami S., Findlay I., and Kurachi Y. 2010 Inwardly rectifying potassium channels: Their structure, function, and physiological roles. Physiol. Rev. 90:291–366. 10.1152/physrev.00021.2009 20086079

[bib16] Hille B., Dickson E.J., Kruse M., Vivas O., and Suh B.C. 2015 Phosphoinositides regulate ion channels. Biochim. Biophys. Acta. 1851:844–856. 10.1016/j.bbalip.2014.09.010 25241941 PMC4364932

[bib17] Ho I.H., and Murrell-Lagnado R.D. 1999 Molecular determinants for sodium-dependent activation of G protein-gated K^+^ channels. J. Biol. Chem. 274:8639–8648. 10.1074/jbc.274.13.8639 10085101

[bib18] Huang C.L., Feng S., and Hilgemann D.W. 1998 Direct activation of inward rectifier potassium channels by PIP2 and its stabilization by Gβγ. Nature. 391:803–806. 10.1038/35882 9486652

[bib19] Humphrey W., Dalke A., and Schulten K. 1996 VMD: Visual molecular dynamics. J. Mol. Graph. 14:33–38. 10.1016/0263-7855(96)00018-5 8744570

[bib20] Inanobe A., Horio Y., Fujita A., Tanemoto M., Hibino H., Inageda K., and Kurachi Y. 1999 Molecular cloning and characterization of a novel splicing variant of the Kir3.2 subunit predominantly expressed in mouse testis. J. Physiol. 521:19–30. 10.1111/j.1469-7793.1999.00019.x 10562331 PMC2269641

[bib21] Jo S., Kim T., and Im W. 2007 Automated builder and database of protein/membrane complexes for molecular dynamics simulations. PLoS One. 2:e880 10.1371/journal.pone.0000880 17849009 PMC1963319

[bib22] Kobayashi T., Ikeda K., Kojima H., Niki H., Yano R., Yoshioka T., and Kumanishi T. 1999 Ethanol opens G-protein-activated inwardly rectifying K^+^ channels. Nat. Neurosci. 2:1091–1097. 10.1038/16019 10570486

[bib23] Lee C.H., Huang P.T., Liou H.H., Lin M.Y., Lou K.L., and Chen C.Y. 2016 Non-basic amino acids in the ROMK1 channels via an appropriate distance modulate PIP2 regulated pHi-gating. Biochem. Biophys. Res. Commun. 473:303–310. 10.1016/j.bbrc.2016.03.100 27016482

[bib24] Lee S.J., Wang S., Borschel W., Heyman S., Gyore J., and Nichols C.G. 2013 Secondary anionic phospholipid binding site and gating mechanism in Kir2.1 inward rectifier channels. Nat. Commun. 4:2786 10.1038/ncomms3786 24270915 PMC3868208

[bib25] Lesage F., Guillemare E., Fink M., Duprat F., Heurteaux C., Fosset M., Romey G., Barhanin J., and Lazdunski M. 1995 Molecular properties of neuronal G-protein-activated inwardly rectifying K^+^ channels. J. Biol. Chem. 270:28660–28667. 10.1074/jbc.270.48.28660 7499385

[bib26] Lewohl J.M., Wilson W.R., Mayfield R.D., Brozowski S.J., Morrisett R.A., and Harris R.A. 1999 G-protein-coupled inwardly rectifying potassium channels are targets of alcohol action. Nat. Neurosci. 2:1084–1090. 10.1038/16012 10570485

[bib27] Logothetis D.E., Kurachi Y., Galper J., Neer E.J., and Clapham D.E. 1987 The β γ subunits of GTP-binding proteins activate the muscarinic K^+^ channel in heart. Nature. 325:321–326. 10.1038/325321a0 2433589

[bib28] Lopes C.M., Zhang H., Rohacs T., Jin T., Yang J., and Logothetis D.E. 2002 Alterations in conserved Kir channel-PIP2 interactions underlie channelopathies. Neuron. 34:933–944. 10.1016/S0896-6273(02)00725-0 12086641

[bib29] Luján R., Maylie J., and Adelman J.P. 2009 New sites of action for GIRK and SK channels. Nat. Rev. Neurosci. 10:475–480. 10.1038/nrn2668 19543219

[bib30] Lüscher C., and Slesinger P.A. 2010 Emerging roles for G protein-gated inwardly rectifying potassium (GIRK) channels in health and disease. Nat. Rev. Neurosci. 11:301–315. 10.1038/nrn2834 20389305 PMC3052907

[bib31] Lüscher C., Jan L.Y., Stoffel M., Malenka R.C., and Nicoll R.A. 1997 G protein-coupled inwardly rectifying K+ channels (GIRKs) mediate postsynaptic, but not presynaptic, transmitter actions in hippocampal neurons. Neuron. 19:687–695. 10.1016/S0896-6273(00)80381-5 9331358

[bib32] Mayfield J., Blednov Y.A., and Harris R.A. 2015 Behavioral and genetic evidence for GIRK channels in the CNS: Role in physiology, pathophysiology, and drug addiction. Int. Rev. Neurobiol. 123:279–313. 10.1016/bs.irn.2015.05.016 26422988 PMC4769645

[bib33] Meng X.Y., Zhang H.X., Logothetis D.E., and Cui M. 2012 The molecular mechanism by which PIP(2) opens the intracellular G-loop gate of a Kir3.1 channel. Biophys. J. 102:2049–2059. 10.1016/j.bpj.2012.03.050 22824268 PMC3341553

[bib34] Meng X.Y., Liu S., Cui M., Zhou R., and Logothetis D.E. 2016 The molecular mechanism of opening the helix bundle crossing (HBC) gate of a Kir channel. Sci. Rep. 6:29399 10.1038/srep29399 27439597 PMC4954981

[bib35] Petit-Jacques J., Sui J.L., and Logothetis D.E. 1999 Synergistic activation of G protein-gated inwardly rectifying potassium channels by the βγ subunits of G proteins and Na(+) and Mg(2+) ions. J. Gen. Physiol. 114:673–684. 10.1085/jgp.114.5.673 10532964 PMC2230539

[bib36] Reuveny E., Slesinger P.A., Inglese J., Morales J.M., Iñiguez-Lluhi J.A., Lefkowitz R.J., Bourne H.R., Jan Y.N., and Jan L.Y. 1994 Activation of the cloned muscarinic potassium channel by G protein β γ subunits. Nature. 370:143–146. 10.1038/370143a0 8022483

[bib37] Rjasanow A., Leitner M.G., Thallmair V., Halaszovich C.R., and Oliver D. 2015 Ion channel regulation by phosphoinositides analyzed with VSPs-PI(4,5)P2 affinity, phosphoinositide selectivity, and PI(4,5)P2 pool accessibility. Front. Pharmacol. 6:127 10.3389/fphar.2015.00127 26150791 PMC4472987

[bib38] Rohács T., Chen J., Prestwich G.D., and Logothetis D.E. 1999 Distinct specificities of inwardly rectifying K(+) channels for phosphoinositides. J. Biol. Chem. 274:36065–36072. 10.1074/jbc.274.51.36065 10593888

[bib39] Scanziani M. 2000 GABA spillover activates postsynaptic GABA(_B_) receptors to control rhythmic hippocampal activity. Neuron. 25:673–681. 10.1016/S0896-6273(00)81069-7 10774734

[bib40] Soom M., Schönherr R., Kubo Y., Kirsch C., Klinger R., and Heinemann S.H. 2001 Multiple PIP_2_ binding sites in Kir2.1 inwardly rectifying potassium channels. FEBS Lett. 490:49–53. 10.1016/S0014-5793(01)02136-6 11172809

[bib41] Sui J.L., Petit-Jacques J., and Logothetis D.E. 1998 Activation of the atrial KACh channel by the βγ subunits of G proteins or intracellular Na+ ions depends on the presence of phosphatidylinositol phosphates. Proc. Natl. Acad. Sci. USA. 95:1307–1312. 10.1073/pnas.95.3.1307 9448327 PMC18753

[bib42] Tao X., Avalos J.L., Chen J., and MacKinnon R. 2009 Crystal structure of the eukaryotic strong inward-rectifier K^+^ channel Kir2.2 at 3.1 A resolution. Science. 326:1668–1674. 10.1126/science.1180310 20019282 PMC2819303

[bib43] Whorton M.R., and MacKinnon R. 2011 Crystal structure of the mammalian GIRK2 K^+^ channel and gating regulation by G proteins, PIP_2_, and sodium. Cell. 147:199–208. 10.1016/j.cell.2011.07.046 21962516 PMC3243363

[bib44] Whorton M.R., and MacKinnon R. 2013 X-ray structure of the mammalian GIRK2-βγ G-protein complex. Nature. 498:190–197. 10.1038/nature12241 23739333 PMC4654628

[bib45] Wickman K.D., Iñiguez-Lluhl J.A., Davenport P.A., Taussig R., Krapivinsky G.B., Linder M.E., Gilman A.G., and Clapham D.E. 1994 Recombinant G-protein β γ-subunits activate the muscarinic-gated atrial potassium channel. Nature. 368:255–257. 10.1038/368255a0 8145826

[bib46] Wickman K., Karschin C., Karschin A., Picciotto M.R., and Clapham D.E. 2000 Brain localization and behavioral impact of the G-protein-gated K^+^ channel subunit GIRK4. J. Neurosci. 20:5608–5615. 10908597 10.1523/JNEUROSCI.20-15-05608.2000 PMC6772558

[bib47] Wiser O., Qian X., Ehlers M., Ja W.W., Roberts R.W., Reuveny E., Jan Y.N., and Jan L.Y. 2006 Modulation of basal and receptor-induced GIRK potassium channel activity and neuronal excitability by the mammalian PINS homolog LGN. Neuron. 50:561–573. 10.1016/j.neuron.2006.03.046 16701207

[bib48] Wu E.L., Cheng X., Jo S., Rui H., Song K.C., Dávila-Contreras E.M., Qi Y., Lee J., Monje-Galvan V., Venable R.M., 2014 CHARMM-GUI Membrane Builder toward realistic biological membrane simulations. J. Comput. Chem. 35:1997–2004. 10.1002/jcc.23702 25130509 PMC4165794

[bib49] Xiao J., Zhen X.G., and Yang J. 2003 Localization of PIP2 activation gate in inward rectifier K+ channels. Nat. Neurosci. 6:811–818. 10.1038/nn1090 12858177

[bib50] Zhang H., He C., Yan X., Mirshahi T., and Logothetis D.E. 1999 Activation of inwardly rectifying K+ channels by distinct PtdIns(4,5)P2 interactions. Nat. Cell Biol. 1:183–188. 10559906 10.1038/11103

[bib51] Zhou W., Arrabit C., Choe S., and Slesinger P.A. 2001 Mechanism underlying bupivacaine inhibition of G protein-gated inwardly rectifying K^+^ channels. Proc. Natl. Acad. Sci. USA. 98:6482–6487. 10.1073/pnas.111447798 11353868 PMC33494

