# Supplementary material for: Dynamic role of the tether helix in PIP2-dependent gating of a G protein–gated potassium channel
Source: J Gen Physiol. 2017 Aug 7;149(8):799–811. doi: 10.1085/jgp.201711801 (PMC5560777; doi:10.1085/jgp.201711801)
Supplement: Supplemental Materials (PDF) [file JGP_201711801_sm.pdf]

SUPPLEMENTAL MATERIAL

Lacin et al., https://doi.org/10.1085/jgp.201711801

Supplemental materials and methods

Cell culture

Human embryonic kidney 293T (HEK293T) cells were cultured in Dulbecco's modified Eagle's medium (Sigma-Aldrich) supplemented with 10% (v/v) FBS, 100 U/ml penicillin, 100 µg/ml streptomycin, and 1× GlutaMAX (Thermo Fisher Scientific) at 37°C and in 5% CO_2 humidified atmosphere. Cell were plated in 12-well plates and transiently transfected with DNA using Lipofectamine 2000 (Thermo Fisher Scientific). Transfected HEK293 cells were replated onto 12-mm glass coverslips for recordings 18 to 42 h later. Cells were transfected with the channel cDNA (0.2 µg GIRK2c, 0.4 µg Kir2.1, 0.2 µg K199C;5′C, 1.6 µg K200C;6′C, 0.2 µg K200F;6′F, 0.2 µg K200Y;6′Y, 0.2 µg K200R;6′R, or 0.2 µg K200S;6′S) and 0.02 µg eYFP cDNA to identify successfully transfected cells. For the PIP_2 depletion experiments, cells were transfected with a voltage-sensitive phosphatase from 0.8 µg zebrafish *Dr-Vsp* cDNA.

Electrophysiology

Whole-cell patch-clamp recordings were made as described previously (Bodhinathan and Slesinger, 2013). Currents were recorded with an Axopatch 200B (Molecular Devices, Axon Instruments) amplifier, compensated electronically for cell capacitance and series resistance (75–100%), filtered at 1 kHz with an 8-pole Bessel filter, and digitized at 5 kHz with a Digidata 1320 interface (Molecular Devices, Axon Instruments). Currents were elicited with voltage ramp protocol, from −120 mV to +50 mV, delivered at 0.5 Hz. Basal K^+ currents (Ba^{2+} sensitive) were quantified at −120 mV by applying 1 mM $BaCl_2$ in the extracellular 20K solution and measuring the amplitude of the current inhibited by Ba^{2+}. The effect of various modulators were measured at −100 mV or −120 mV by averaging current amplitudes from at least three consecutive sweeps at steady state and subtracting the mean basal current before and after application of the modulator. For studying the effect of depleting membrane PIP_2, *Dr-Vsp* was activated by a voltage step from a holding potential of −40 mV to +100 mV for varying lengths of time, and the amplitude of the GIRK currents were then measured at −120 mV. This three-step protocol was repeated every 2 seconds (i.e., *Dr-Vsp* activation protocol; Bodhinathan and Slesinger, 2013). The extent of *Dr-Vsp* inhibition was calculated as a fraction of the Ba-sensitive basal current. The decrease in current (i.e., depletion) and recovery of current following *Dr-Vsp* activation were fit with a single exponential to determine the tau.

MD simulations

The crystal structure of GIRK2 channel (PDB: 4KFM) bound to a PIP_2 head group was used. We used the PDB: 4KFM structure for simulations, because this channel is in a preopen conformation (Whorton and MacKinnon, 2013). We compared the 3SYA and 4KFM structures, and we determined there is a small RMSD (Whorton and MacKinnon, 2011, 2013), with the largest movements in the LM loop and a 0.41 Å RMSD for the c-alpha in the PIP_2 pocket, formed by residues 88 to 92 (from the slide helix), 192 to 203 (HBC gate and tether helix), and 62 to 64 (N-terminal b-loop). Four 1-stearoyl-2-arachidonoyl-*sn*-glycero-3-phosphoinositol 4,5 bisphosphate (18:0, 22:4 PIP_2) molecules were aligned to the crystal structure of PIP_2, one at each binding site. Gβγ subunits were not included in the calculations. The GIRK2-PIP_2 system was embedded in a bilayer consisting of POPC (16:0, 18:1 PC) lipids using the CHARMM-GUI membrane builder (Brooks et al., 2009; Wu et al., 2014). The protein–lipid system was then solvated with TIP3P water molecules and 150 mM KCl. The CHARMM36 force field was employed (Best et al., 2012). Additionally, five potassium ions were placed in the ion conduction pathway and four sodium ions were placed in the sodium-binding site as seen in the crystal structures. A second system was set up with the same conditions but with a 6′Y mutation generated by in silico mutagenesis using CHARMM-GUI. The two systems (WT and 6′Y) were subjected to stepwise decreased restraint equilibration used in CHARMM-GUI membrane builder (Jo et al., 2007). The two systems (WT and 6′Y) were then equilibrated for 20 ns using positional restrain of 1,000 $KJ/mol/nm^2$ on all Cα atoms of the protein before the 400-ns unrestrained production simulations. We then repeated the 400-ns production simulations using different initial velocities for both systems.

Simulations were conducted using GROMACS 4.6 (Hess et al., 2008) with a 2-fs integration time step. A periodic boundary condition was applied in all directions, and the temperature and semi-isotropic pressure with a compressibility of 4.5×10^{-5} bar^{-1} were controlled by a velocity-rescale coupling algorithm and Parrinello-Rahman barostat (Bussi et al., 2007). The electrostatic interactions were calculated using the particle mesh Ewald with 0.12-nm Fourier spacing, and LINCS constraints were applied to all bonds (Hess et al., 1997). All repeat simulations were performed with differences in initial velocity.

Simulations were visualized using VMD, Pymol (Humphrey et al., 1996), or Discovery Visualizer 4.0 (Accelrys). Hydrogen bond analysis was conducted using GROMACS g-hbond with default cutoff of 3.5 Å and 30° between hydrogen bond donors (OH and NH) and acceptor oxygens. The probability of hydrogen bonds was determined by dividing the total number of hydrogen bonds by the simulation time. The relative PIP_2 association was calculated by taking the sum of hydrogen bonds for 0′K–5′-PO_4, 5′K–5′-PO_4, and 6′K–5′-PO_4 and subtracting hydrogen bonds for the non-PIP_2 association (e.g., K200-E203). Cross-distance diameter of F192 gate was calculated by measuring the distance of the center of mass of the F192 (−2′F) side chain between chain A–C and chain B–D and averaging the two. Water analysis was conducted by counting number of water in 15 × 15–Å box along the pore axis of the channel. Probability density of water was obtained by calculating water count at the pore throughout the 400 ns simulation and normalizing to the probability density of water at the bottom of the selectivity filter.

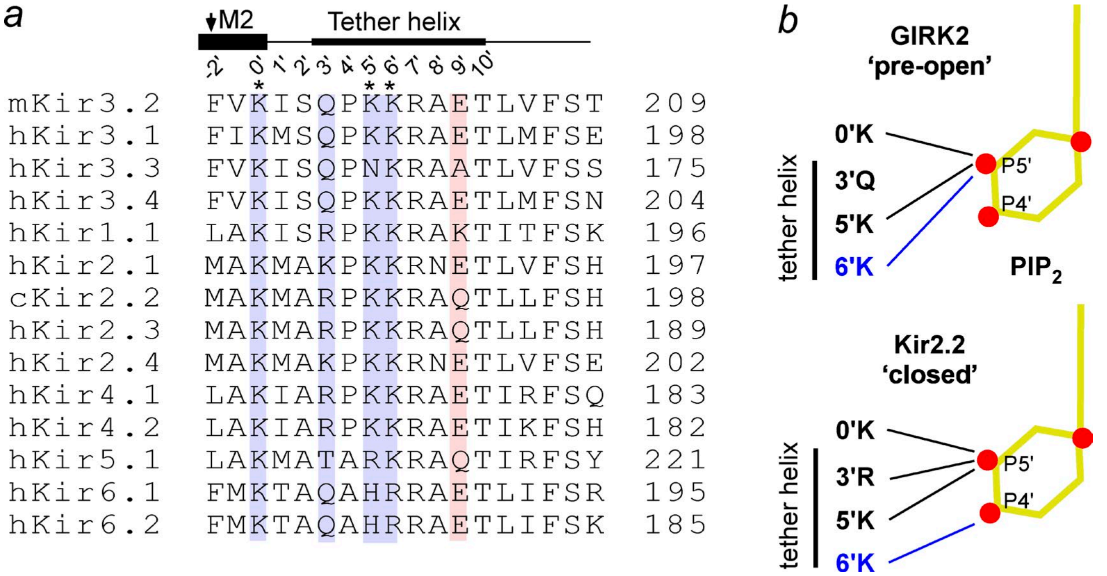

Figure S1. **Alignment of amino acids in the tether helix.** (a) Sequence alignment for all members of the Kir channel family. Note the numbering scheme, where K_{194} is the 0′ position. Arrow indicates gate in the HBC, F192 (−2′F). (b) Comparison of hydrogen bonds between basic residues in the tether helix and PIP_2, based on the GIRK2/PIP_2 (PDB: 4KFM) and Kir2.2/PIP_2 structures (PDB: 3SPI; Hansen et al., 2011; Whorton and MacKinnon, 2011, 2013).

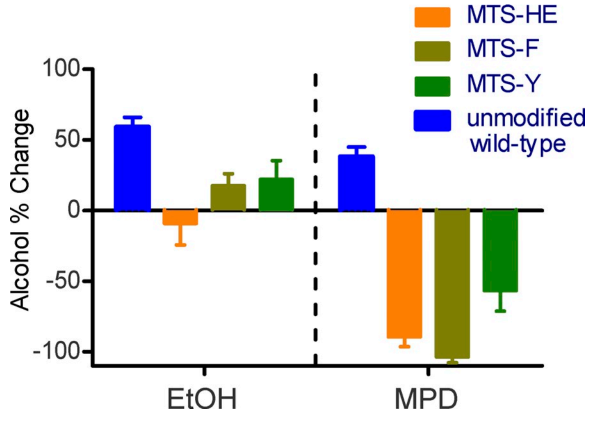

Figure S2. **Mean percentage change in basal current with alcohol (EtOH, MPD) and GIRK2*, GIRK2*+MTS-HE, GIRK2*+MTS-F, or GIRK2*+MTS-Y.** Note inhibition by MPD for all three modified channels. Bars represent mean ± SEM.

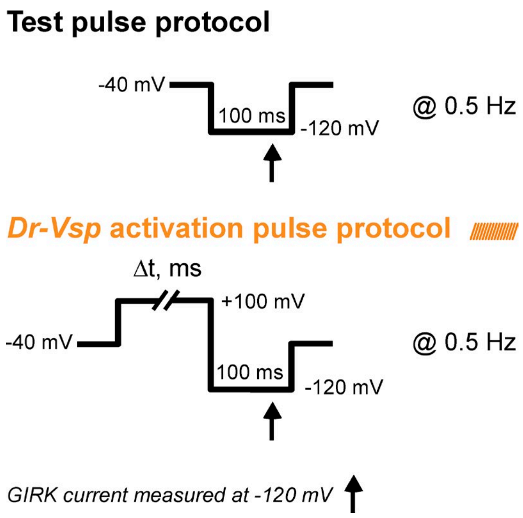

Figure S3. **Voltage protocol used to activate Dr-Vsp.** Baseline current is measured at −120 mV with the test pulse protocol. Dr-Vsp is activated by voltage steps to +100 mV for varying length of time (Δt, milliseconds), and then GIRK current is measured at the end of the voltage step to −120 mV (Dr-Vsp activation protocol). Pulses are delivered at 0.5 Hz.

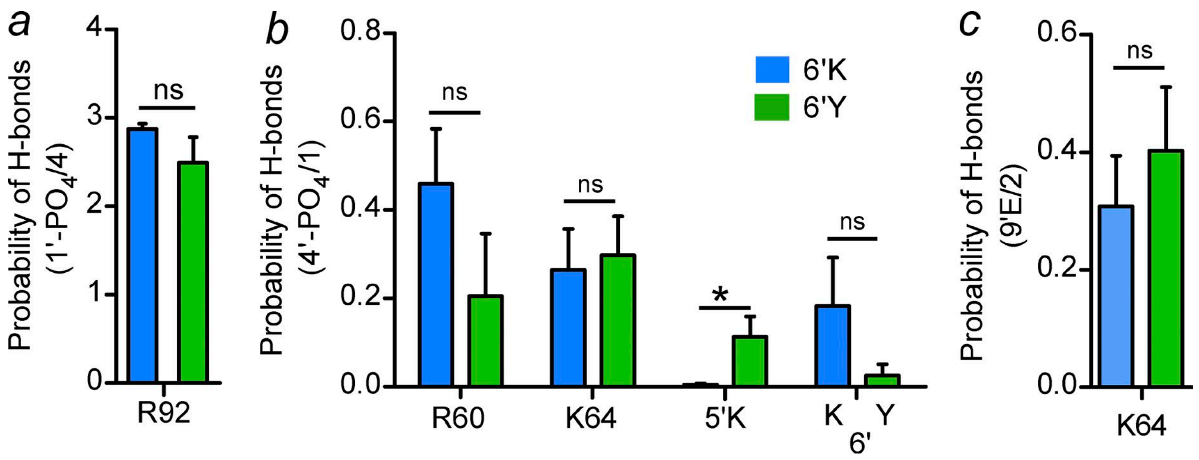

Figure S4. **Probability of hydrogen bond formation for 1'-PO_4, 4′-PO_4, and K64-9′E.** (a) Probability of hydrogen bonds for R92 and 1′-PO_4 of PIP_2 (of 4 maximal bonds). (b) Probability of hydrogen bonds for 4′-PO_4 and R60, K64, 5′K and 6′K in the 6′K-WT and 6′Y simulations. (c) Probability of hydrogen bonds for 9′E and K64 for 6′K-WT and 6′Y simulations. Bars represent mean ± SEM. *, $P < 0.05$ using Student's *t* test.

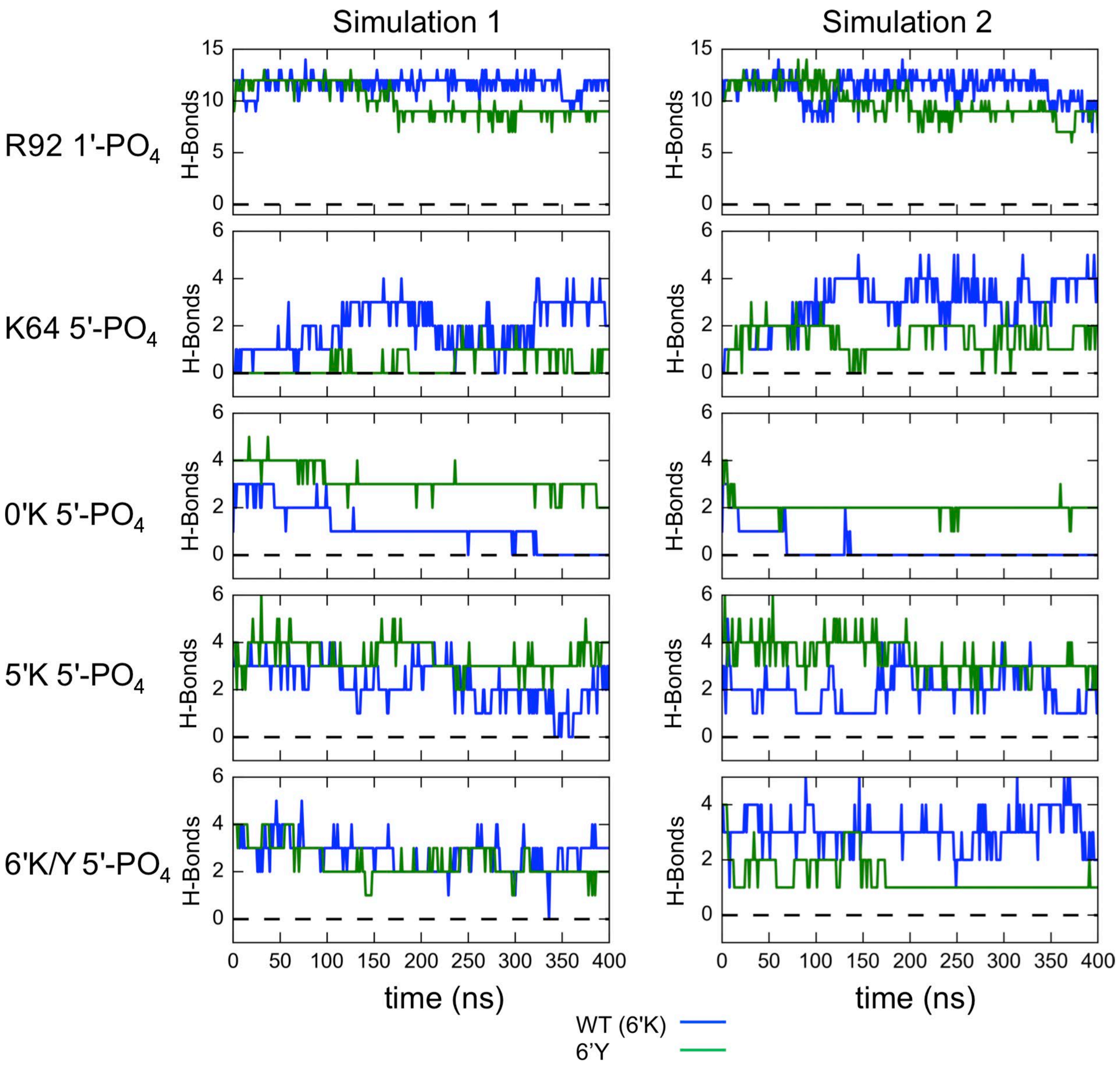

Figure S5. **Time dependence of hydrogen bonds at all four PIP_2-binding sites during two 400-ns simulations for GIRK2-6′K (blue) and GIRK2-6′Y (green).** Note the scale bar on 1′-PO_4 interaction with R92 interaction, indicating the strong and relatively stable interaction of PIP_2 with the channel at the 1′-PO_4 position.

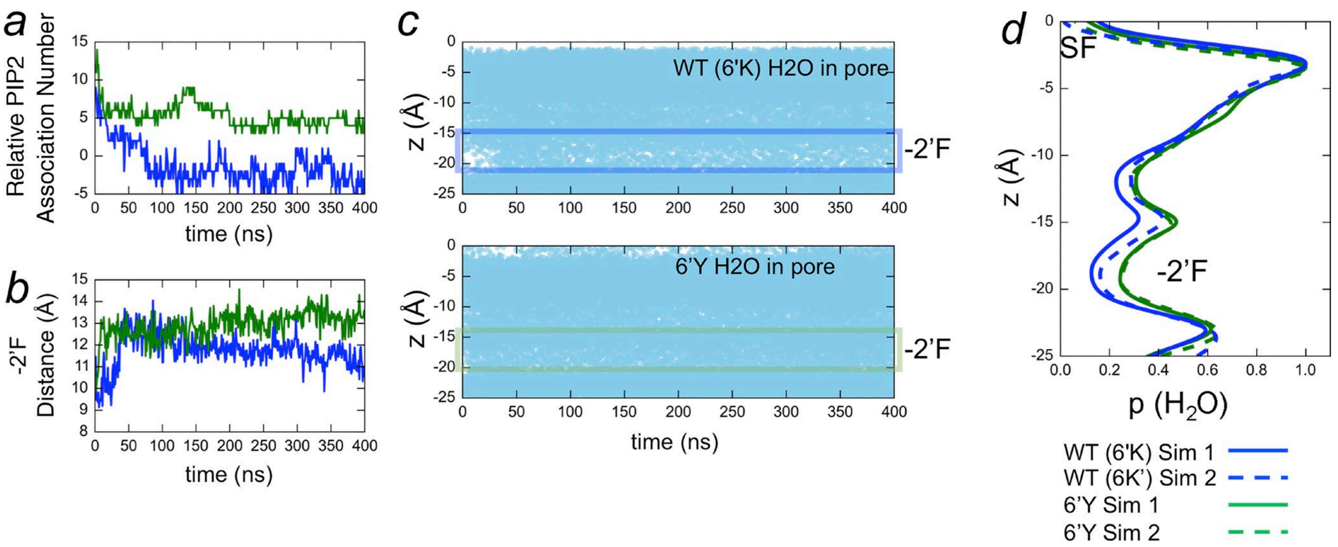

Figure S6. **Data for simulation 2 show a trend similar to simulation 1 in Fig. 6.** (a) PIP_2 association number, (b) −2′F cross-distance, and (c) water profile of simulation 2. (d) Probability density of water for each simulation shows that WT has a decreased water density at the −2′F gate in both simulations compared with 6′Y mutant simulations. SF, selectivity filter.

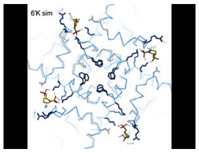

Video 1. **Video of the MD simulation of WT GIRK2 at the level of the HBC.** Note the movement of the F192 in the HBC, as well as in PIP_2.

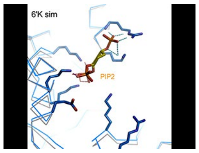

Video 2. **Video of the 400-ns MD simulation of the PIP_2 pocket in one subunit of the WT channel.** All simulations are for 400 ns. The trajectories are 1-ns snapshots that were fit progressively on the c-α atoms using trjconv command in Gromacs. The trajectories were smoothened once using the “smooth” command in Pymol to suppress high-frequency vibrations to visualize the videos.

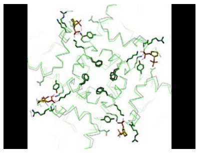

Video 3. **Video of the MD simulation of GIRK2 6′Y at the level of the HBC.** Note the movement of the −2′F (F192) in the HBC, as well as in PIP_2.

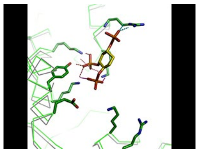

Video 4. **Video of the 400-ns MD simulation of the PIP_2 pocket in one subunit of the GIRK2 6′Y channel.** All simulations are for 400 ns. The trajectories are 1-ns snapshots that were fit progressively on the c-α atoms using trjconv command in Gromacs. The trajectories were smoothened once using the “smooth” command in Pymol to suppress high-frequency vibrations to visualize the videos.

REFERENCES

Best, R.B., X. Zhu, J. Shim, P.E. Lopes, J. Mittal, M. Feig, and A.D. Mackerell Jr. 2012. Optimization of the additive CHARMM all-atom protein force field targeting improved sampling of the backbone φ, ψ and side-chain χ(1) and χ(2) dihedral angles. *J. Chem. Theory Comput.* 8:3257–3273. http://dx.doi.org/10.1021/ct300400x

Bodhinathan, K., and P.A. Slesinger. 2013. Molecular mechanism underlying ethanol activation of G-protein-gated inwardly rectifying potassium channels. *Proc. Natl. Acad. Sci. USA.* 110:18309–18314. http://dx.doi.org/10.1073/pnas.1311406110

Brooks, B.R., C.L. Brooks III, A.D. Mackerell Jr., L. Nilsson, R.J. Petrella, B. Roux, Y. Won, G. Archontis, C. Bartels, S. Boresch, et al. 2009. CHARMM: The biomolecular simulation program. *J. Comput. Chem.* 30:1545–1614. http://dx.doi.org/10.1002/jcc.21287

Bussi, G., D. Donadio, and M. Parrinello. 2007. Canonical sampling through velocity rescaling. *J. Chem. Phys.* 126:014101. http://dx.doi.org/10.1063/1.2408420

Hansen, S.B., X. Tao, and R. MacKinnon. 2011. Structural basis of PIP2 activation of the classical inward rectifier K+ channel Kir2.2. *Nature.* 477:495–498. http://dx.doi.org/10.1038/nature10370

Hess, B., H. Bekker, H.J.C. Berendsen, and J.G.E.M. Fraaije. 1997. LINCS: A linear constraint solver for molecular simulations. *J. Comput. Chem.* 18:1463–1472. http://dx.doi.org/10.1002/(SICI)1096-987X(199709)18:12<1463::AID-JCC4>3.0.CO;2-H

Hess, B., C. Kutzner, D. van der Spoel, and E. Lindahl. 2008. GROMACS 4: Algorithms for highly efficient, load-balanced, and scalable molecular simulation. *J. Chem. Theory Comput.* 4:435–447. http://dx.doi.org/10.1021/ct700301q

Humphrey, W., A. Dalke, and K. Schulten. 1996. VMD: Visual molecular dynamics. *J. Mol. Graph.* 14:33–38. http://dx.doi.org/10.1016/0263-7855(96)00018-5

Jo, S., T. Kim, and W. Im. 2007. Automated builder and database of protein/membrane complexes for molecular dynamics simulations. *PLoS One.* 2:e880. http://dx.doi.org/10.1371/journal.pone.0000880

Whorton, M.R., and R. MacKinnon. 2011. Crystal structure of the mammalian GIRK2 K^+ channel and gating regulation by G proteins, PIP_2, and sodium. *Cell.* 147:199–208. http://dx.doi.org/10.1016/j.cell.2011.07.046

Whorton, M.R., and R. MacKinnon. 2013. X-ray structure of the mammalian GIRK2-βγ G-protein complex. *Nature.* 498:190–197. http://dx.doi.org/10.1038/nature12241

Wu, E.L., X. Cheng, S. Jo, H. Rui, K.C. Song, E.M. Dávila-Contreras, Y. Qi, J. Lee, V. Monje-Galvan, R.M. Venable, et al. 2014. CHARMM-GUI Membrane Builder toward realistic biological membrane simulations. *J. Comput. Chem.* 35:1997–2004. http://dx.doi.org/10.1002/jcc.23702
